# Wind turbine with line-side PMSG FED DC-DC converter for voltage regulation

**DOI:** 10.1371/journal.pone.0305272

**Published:** 2024-06-28

**Authors:** B. Nagi Reddy, Radhika Jalli, K. Sai Prudhviraj, K. Badrinath Shetty, Ch. Rami Reddy, Hossam Kotb, Ahmed Emara, Mohammed Alruwaili

**Affiliations:** 1 Department of Electrical and Electronics Engineering, Vignana Bharathi Institute of Technology Hyderabad, Hyderabad, India; 2 Department of Electrical and Electronics Engineering, Joginpally B R Engineering College, Hyderabad, India; 3 Applied Science Research Center, Applied Science Private University, Amman, Jordan; 4 Department of Electrical Power and Machines, Faculty of Engineering, Alexandria University, Alexandria, Egypt; 5 Electrical Engineering Department, University of Business and Technology, Jeddah, Saudi Arabia; 6 Department of Electrical Engineering, College of Engineering, Northern Border University, Arar, Saudi Arabia; HJNU: Hanjiang Normal University, CHINA

## Abstract

This article represents a novel study of the design and analysis of a wind turbine system that includes a line-side permanent magnet synchronous generator (PMSG) with an ultra-step-up DC-DC converter for voltage regulation. Integrating renewable energy sources such as wind power into the grid requires efficient and reliable power conversion systems to handle fluctuating power and ensure a stable power supply. The wind turbine system utilizes a PMSG, which offers several advantages over traditional induction generators, including higher efficiency, reduced maintenance, and better power quality. The line-side configuration allows for increased control and flexibility, allowing the system to respond dynamically to grid conditions. This wind turbine system involves the integration of a grid-side PMSG-fed DC-DC converter between the PMSG and the grid. The converter enables a seamless flow of electricity between the wind turbine and the grid. By actively controlling the intermediate circuit voltage, the converter efficiently regulates the output voltage of the wind turbine and thus enables constant power generation regardless of fluctuating wind speeds. The simulation outcomes illustrate the efficacy of the proposed system in achieving voltage regulation and seamless integration with the grid. Performance is evaluated under various operating conditions and compared to conventional wind turbines.

## 1. Introduction

Renewable energy sources have received much attention recently due to the depletion of fossil fuel supplies and the rise in greenhouse gas emissions [1]. Wind energy is one of the most environmentally friendly power sources and has the speediest development [2, 3]. The energy remains sustainable as long as the sun continues to heat the planet. Over the decades, the prevalence of renewable energy from wind power has steadily increased [4]. According to a statistical analysis by the International Renewable Energy Agency (IRENA) global installed wind power production capacity, including onshore and offshore, has grown by a factor of 98 over the past 20 years, ascending from 7.5 GW in 1997 to roughly 733 GW in 2018 [5–8]. The limit of energy coastal Breeze energy expanded from 178 GW in 2010 to 699 GW in 2020, while the seaward wind limit expanded relatively more, though from a lower base, from 3.1 GW in 2010 to 34.4 GW in 2020. Wind energy creation expanded by a variable of 5.2 to 1412 TWh, somewhere in the range between 2009 and 2019 [9–13]. The capacity of wind turbines has improved over time. In the past, standard turbines featured a rated capacity of 0.05 MW and a rotor diameter of 15 meters [14, 15]. Today’s new wind power projects range from 3 to 4 MW onshore and 8 to 12 MW offshore [16].

A widely used system in wind turbines is the Doubly-Fed Induction Generator (DFIG) with the back-to-back converter. In a DFIG, the generator’s stator is connected directly to the grid [17]. The rotor is connected to the grid via a back-to-back power converter. This variable-speed design is typically used in the 1.5 MW and 6 MW power range. In a DFIG converter, power flow through the power semiconductor is limited to one-third in both directions [18].

In contrast to the DFIG wind turbine, The PMSG wind turbine is used in wind energy conversion, has a higher efficiency and power factor, requires no routine maintenance, and has flexible active and reactive power control. This approach is renowned for its enhanced energy efficiency and reduced mechanical strain [19, 20]. Power conditioning is being significantly impacted by the use of power electronics converters (PECs) in power system applications, notably in renewable energy systems connected to the electrical grid. The topology with back-to-back converters is the most alluring setup for PMSG-based wind energy conversion systems (WECSs) [21–23]. Additionally, recent advancements in power electronics and control methodologies have expanded the possibilities for voltage regulation within PMSG-based wind turbine systems. Synchronous generators produce the majority of the electricity used in commercial. It is standard practice to employ them to transform the mechanical power output of steam turbines, gas turbines, reciprocating engines, and hydro turbines into electrical power for the grid [24].

An electrical device known as a DC-DC converter is a type of power converter that changes the voltage level of a direct current (DC) source. Before the invention of power semiconductors, one method for raising the voltage of a DC supply for low-power applications involved converting it to AC first using a vibrator, then a step-up transformer, and finally using a rectifier [25]. Nowadays, DC-DC converters are pivotal components across a diverse range of applications, encompassing motor control, switch-mode DC power supplies (SMPS), Uninterruptible Power Supplies (UPS), Regenerative Braking Systems (RBS), Battery Storage Systems, wind farms, and photovoltaic (PV) cells. In wind farm setups focused on DC power collection, the need arises for high-capacity MW-level DC-DC converters to elevate the relatively low DC voltage sourced from the integrated rectifier of wind generators to a higher DC voltage suitable for efficient transmission to the mainland. Consequently, a DC-DC converter assumes a central role within High Voltage Direct Current (HVDC) systems aimed at seamlessly integrating wind farms into the grid [26]. A fifth-order buck converter is also a type of DC-DC converter. This converter exhibits improved bucking characteristics at moderate duty ratios through time-domain analysis of steady-state converter performance.

Building on the research, this paper introduces a novel non-isolated step-up DC-DC converter suitable for low-voltage distributed power systems in both standalone and grid-connected applications. Presently, there are two types of Insulated Gate Bipolar Transistor (IGBT) switches available: one with a voltage rating of 4.5 kV and a maximum nominal current of 2400 A, and the other with a voltage rating of 6.5 kV and a maximum nominal current of 750 A. However, the latter option comes at a higher cost. Consequently, the operational voltage level poses a significant challenge in many research endeavors, as both the input and output of the DC-DC converter must function at levels exceeding the voltage capacity of existing power semiconductor devices. The proposed converter offers several advantages over other converters. It is necessary to step up the Voltage Boost converters because they can increase the input voltage to a desired amount while staying within realistic bounds and using minimal parts [27, 28]. A Boost converter is a DC-DC converter with an output voltage more significant than its source voltage. The primary focus of this innovation is enhancing the boost factor and efficiency while concurrently reducing the component count and electrical stress on power components, maintaining a continuous source current, and reducing source current ripple, addressing key challenges in designing such converter topologies. However, it does have a limitation on its maximum achievable voltage gain [29, 30]. These high step-up converters were developed by integrating quadratic boost and Cuk converter concepts.

## 2. Literature survey

In this paper [24], the performance of PMSG-based WECs relative to conventional induction generator-based WECS is the primary emphasis of this article. It focuses primarily on contrasting the PMSG-based WECs’ parameters with those of other WECs. Discussing how to design PMSG-based WECs to satisfy the grid codes’ requirements for low voltage. [25] This survey investigates a step-up interleaved DC-DC converter (IDC) operating in three distinct duty regions (0 ≤ *D* < 0.5, 0.5 ≤ *D* < 1, and 0 ≤ *D* < 1) with reduced component count. The study encompasses comprehensive mathematical modeling of the IDC across these regions and compares it with existing converters. It discusses advantages and drawbacks and evaluates factors such as component count, voltage gain, voltage stresses, input current characteristics, and output voltage nature. Furthermore, a laboratory prototype validates IDC performance, and power loss and efficiency analyses underscore its effectiveness. [26] This paper introduces two innovative non-isolated DC-DC converters tailored for electric vehicle applications, extendable to other electric vehicle types. These converters merge the key attributes of quadratic Boost and Cuk converters, providing high step-up voltage capabilities and simplified control using a single ground-referenced active power switch. Furthermore, the proposed topologies reduce voltage stress on the active power switch in comparison to conventional boost converters. The paper also discusses the design considerations for these converters. To substantiate their effectiveness and feasibility for fuel cell electric vehicles, experimental results from a laboratory prototype are presented, confirming their suitability for real-world applications.

This study demonstrates the excellent performance of the quadratic step-up boost converter topology [27]. The quadratic step-up boost converter is also described in detail, which achieves efficiencies of 96.1%, 95.5%, and 94.3% with voltage gains of 12, 20, and 50 times, respectively. [28] This paper is focused on hybrid, control system, and generator optimization. According to this study, the number of poles in a PMSG relies on the wind speed and the required power output. [29] In this survey introduces an innovative approach to developing an ultra-gain DC-DC converter with minimal componentry. The proposed converter leverages a switched capacitor-inductor network (SCLN) configuration and utilizes a single switch to achieve this objective efficiently. The converter achieves remarkable voltage gain by merging switched-inductor and switched-capacitor principles within a single topology. The paper also emphasizes the stability of the SCLN converter through an analysis of pole positions in its small-signal model. Experimental results validate the converter’s capabilities, showcasing its suitability for both three-phase (650V) and single-phase (325V) applications, particularly in solar photovoltaic systems.

This paper delves into synthesizing a converter family based on a specified conversion ratio using the Flux Balance Principle. It initially explores the relationship between voltage conversion ratio and flux balance equations and derives generalized gains for first and second-order converters. The study introduces an innovative approach for solving the inverse problem, allowing the derivation of a converter topology from a predefined conversion ratio using flux balance principles [30]. A systematic procedure for applying this method is outlined. The study applies this approach to synthesize various quadratic buck-boost converter topologies to demonstrate its effectiveness.

Furthermore, the experimental validation of two newly identified topologies reaffirms the practical utility of the proposed theory. This paper introduces two cascaded buck-boost converters, each tailored for specific voltage transformation requirements and excels at achieving high step-up gains. At the same time, the other specializes in high step-down gains. These converters offer a unique advantage by enabling two distinct duty ratios for a given voltage gain, a feature particularly beneficial for voltage-sensitive applications such as LED systems. The paper provides a comprehensive overview of the operating principles, steady-state analysis, and passive component design for both switching schemes of the proposed converters. Comparative analysis against competing converters underscores the superior performance of the proposed converters in terms of voltage gain and output continuity [31]. Experimental validation of these converters, employing both simultaneous and complementary switching schemes, confirms their theoretical steady-state performance. This comprehensive review presents a novel non-isolated buck-boost DC-DC converter that offers a wide range of conversion ratios. Unlike traditional counterparts, this converter maintains a continuous input current and boasts high step-up voltage gain, rendering it suitable for industrial and renewable energy applications. The paper comprehensively explains the converter’s operational principles and offers a detailed small-signal model [32]. It conducts a comparative analysis with other buck-boost topologies, considering factors such as the number of elements, voltage gain, switch stress, and input current type (continuous or discontinuous). Emphasizing its applicability in renewable energy systems, particularly photovoltaic setups, the paper substantiates its findings through experimental validation via a prototype.

In this paper conducts a systematic and analytical exploration of non-isolated Reduced Redundant Power Processing (*R*^*2*^*P*^*2*^) converter topologies. It derives all feasible non-isolated *R*^*2*^*P*^*2*^ configurations and offers detailed examples to elucidate various scenarios. The study calculates voltage ratios and efficiencies for each *R*^*2*^*P*^*2*^ configuration, emphasizing high step-up voltage gain and efficiency for high-power applications. Among these configurations, one is singled out for further examination as the most suitable for such applications. The paper then compares theoretical analyses and calculations of step-up voltage ratios and efficiency with experimental results from a 2kW laboratory prototype, validating the effectiveness of this investigation [33]. The paper explores a Switched Capacitor-Inductor Network (SCLN) based DC-DC converter, emphasizing its detailed analytical waveforms in both Continuous Conduction Mode (CCM) and Discontinuous Conduction Mode (DCM). The survey highlights accurate efficiency measurements considering parasitic elements, derivation of a small-signal model, and experimental validation, demonstrating the converter’s potential merit in single-phase and three-phase solar PV applications [34]. Notably, it confirms the practical operation of the SCLN converter with an impressive maximum gain of 25.83.2. This paper concisely overviews a novel buck-boost DC-DC converter with semi-quadratic voltage gain. The converter offers several advantages, including continuous input current, common ground, low ripple input current, positive output voltage, and large voltage gain. Furthermore, it minimizes voltage stresses across power switches, enabling the use of low-on-resistance components for improved efficiency [35]. The article thoroughly covers performance principles, DC analysis, modelling, power loss analysis, and practical implementation through a laboratory prototype, making it a valuable contribution to the field of DC-DC converters for carbon-free power sources.

This study introduces a novel high step-up DC-DC converter, incorporating a quadratic boost converter and a voltage multiplier cell. The converter’s design and implementation are based on its core equations, with experimental validation demonstrating strong alignment with theoretical analysis. The research examines key performance factors including voltage stress, voltage gain, efficiency, dynamic behavior, and transient operation, highlighting the converter’s potential for applications requiring significant voltage enhancement [36]. This article introduces a novel method for component selection to design innovative DC-DC converter topologies. The method enhances the famous series-shunt structure, resulting in a family of fourth-order quadratic DC-DC buck-boost converters. These converters have two synchronized active switches and two diodes, offering a broad conversion ratio range. Among these four converters, two exhibit remarkable high step-up gains suitable for renewable energy and electric vehicle applications, while the other two excel in providing high step-down voltage gain, catering to portable electronic devices and integrated circuits [37]. These proposed converters are designed to minimize electric stress on power semiconductor devices, reducing power losses and enhancing efficiency. Additionally, it conducts a comparative assessment against other buck-boost converters, considering factors like voltage gain, electric stress on power semiconductor devices, input-output continuity, common ground, and output polarity.

Experiments are carried out on a 100-W laboratory prototype to validate their performance. This research suggests a non-isolated converter topology to address the unpredictable output voltage characteristics. A broad input voltage range, high voltage gain, low component voltage stress, and construction where the output and input are common ground are some of the advantages of the proposed converter’s electrical performance [38]. The working principle is examined in this paper. Building a small-signal model is the first step in creating the closed-loop control system. Additionally, a prototype is created when the device parameters are calculated. According to the testing findings, the prototype’s input range is 25–60 V. The boost ratio may reach 8 times when the rated output voltage is 200 V, which satisfies the criteria for fuel cell cars for DC/DC converters.

In this paper introduces a novel DC-DC switched-capacitor converter designed to elevate the low voltage output of renewable energy systems to a higher bus voltage, suitable for downstream DC-AC grid-connected inverters. The converter offers the potential for output voltage regulation and provides operational principles with specific switching conditions. The topology enables high voltage gain, and excellent output regulation, and facilitates applications like maximum power point tracking (MPPT) [39]. Notably, it maintains continuous input current, accommodates various DC voltage source values, and offers cost advantages compared to conventional structures. The paper includes a comparative analysis with similar structures and presents experimental results to validate the theory and feasibility of the proposed converter. This comprehensive literature review assesses various unidirectional multistage DC-DC converter families to enhance Tank-To-Wheel (TTW) efficiency in Fuel Cell Vehicles (FCVs), with a focus on cost-efficiency, eco-friendliness, zero emissions, and high power. It categorizes fuel cells based on temperature and power capabilities for FCV selection [40]. Furthermore, it classifies DC-DC converters into different families, such as SCBC, SIBC, Transformer and Coupled Inductor Based Converter, Luo converter, Multilevel converter, and X-Y converter, considering their types and characteristics. Each converter family is evaluated for its pros and cons. The review highlights the recurring use of switched inductors and boosting techniques to enhance performance. Notably, HSI-SC, multilevel, and X-Y converters emerge as favourable choices for FCV applications based on cost and efficiency. The study offers detailed insights into individual converters through comparative analyses, aiding the selection of the best-suited converter for powering the FCV and supporting its luxurious loads.

This paper introduces a novel approach for optimizing power extraction from a fuel cell (FC) stack array by employing dedicated power converters (DPCs) for each FC stack, coupled with a Maximum Power Point Tracking (MPPT) controller. Notably, the paper suggests operating them at the maximum fuel efficiency point (MFEP) instead of the MPPT to prevent potential performance degradation in underperforming FC stacks. This MFEP operation ensures efficient use of reactant gases and maintains safe voltage levels. The DPCs employ phase-shifted converters to enhance conversion efficiency [41]. The scheme’s effectiveness is thoroughly examined through Simulink simulations and confirmed with experimental results, underscoring its practical utility. In this paper presents a novel common ground DC-DC converter designed to meet the specific needs of fuel cell stack power interface converters in fuel cell vehicles. The converter employs an interleaved structure on the input voltage side to minimize input current ripple. On the output voltage side, it incorporates two series capacitors and a diode-capacitor configuration to enhance voltage gain and reduce stress on power semiconductors and capacitors, thereby improving efficiency and reliability [42]. The paper comprehensively addresses the operating principles, steady-state characteristics, parameter design, and efficiency calculations, and compares it with other converter types. To validate the analytical findings, a 1.6 kW prototype with a 400 V output voltage is developed, demonstrating that the proposed converter meets the requirements of a fuel cell stack power interface converter and is well-suited for integration into fuel cell vehicles. [43] a novel non-isolated negative output buck-boost converter topology for wide voltage conversion applications is proposed. It uses a typical buck-boost converter configuration, but replaces the active switches with two switches-two inductors (2S2L) cells. This design allows for a wide range of step-down and step-up voltage conversions. The performance of the proposed system is designed in MATLAB/SIMULINK, and comparisons are presented to demonstrate its competitiveness with other buck-boost converters. The paper [44] proposes a Quasi Z-Source Indirect Matrix Converter (QZSIMC) for Permanent Magnet Generator (PMG) based Direct Drive Wind Energy Conversion System (WECS). The QZSIMC is integrated with an LC filter to reduce harmonic distortion and improve power quality. The results show that the proposed converter can achieve a high power factor, low total harmonic distortion, and high efficiency. The proposed converter can be used for grid-connected and standalone WECS applications.

## 3. Methodology

**Fig 1:**
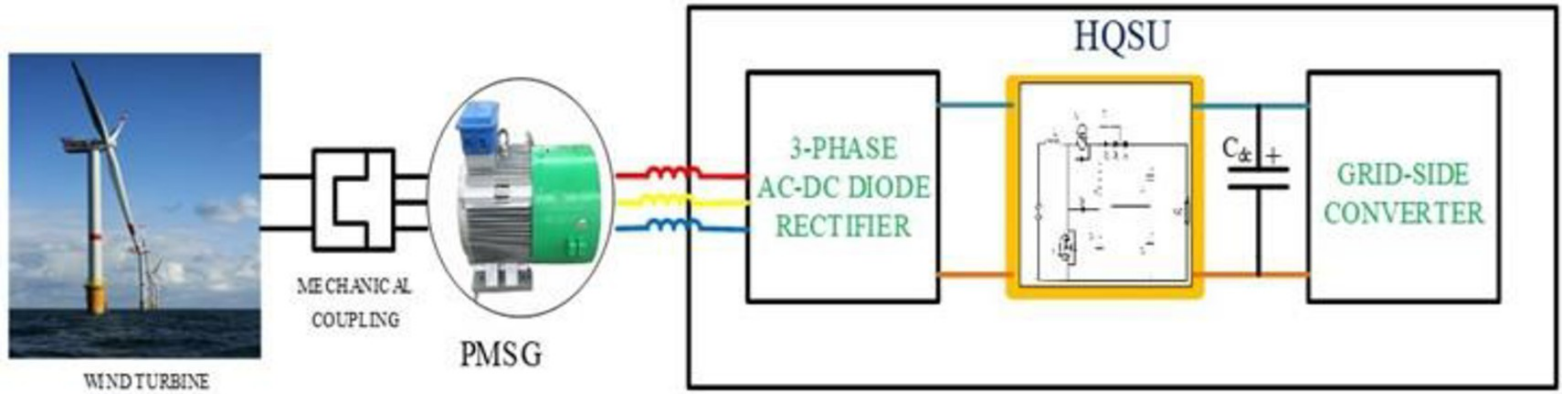
Schematic diagram of line side grid-connected wind turbine with PMSG.

A microgrid is depicted schematically in Fig 1 and consists of a wind turbine (which serves as a DER), a PMSG, a 3-phase AC-DC Diode rectifier, and an HQSU converter. With the aid of a generator, a wind power conversion system (WPCS) converts wind kinetic energy into mechanical energy, which is then converted into electric energy. There are various types of generators used for wind turbines, but PMSG is preferred over induction generators for several reasons, including:

- torque-producing stator current,
- no magnetizing currents,
- higher reliability,
- the absence of the need for gearboxes.

An effective power electronic interface (PEI), consisting of numerous types of converters and associated management and protection systems, is necessary to integrate a prime-mover-based DER, such as PMSG, into the traditional grid.

The various machine-side converters (MSC) and grid-side converters (GSC) are utilized for wind turbines. For the DER, the PEI offers a variety of capabilities, including

- voltage control
- VAR support
- fault ride-through
- power quality standard implementation.

To connect various sheddable and non-sheddable loads to the grid, the grid side converter feeds the grid with active power, which is then stepped up to 480V and provided to the LV bus. To transform the PMSG voltage into an unregulated DC voltage, a 3-phase 6-pulse diode rectifier is employed. Step-up converters are further used to control the DC voltage and convert it to a constant DC-link voltage.

The fundamental Eq (1) for a wind turbine is:

$$P=\frac{1}{2}A\rho V^{3}$$ (1)

A wind turbine’s output is determined by three factors: the air’s density (typically 1.2 kg/m^3^), the swept area of its blades (imagine the blades whirling in a large circle), and the wind’s speed. Wind speed is undoubtedly the factor that varies the most among these. In contrast to the other inputs, wind speed is cubed, making it the variable with the greatest influence. where *V* is the velocity, *ρ* is the air density.

The captured mechanical power of a wind turbine is expressed as:

$$P_{m}=\frac{1}{2}\pi \rho_{ar}V_{v}^{3}C\rho (\alpha ,\beta )$$ (2)

Here *C*ρ represents the wind turbine power coefficient.

The mechanical torque of a wind turbine is given as:

$$\tau_{m}=\frac{1}{2\lambda }\pi R_{1}^{3}\rho_{ar}V^{2}C$$ (3)

The mathematical model of the PMSG machine is most frequently represented by the dynamic (d-q) (synchronous) rotating reference frame. Following the necessary rotational transformations, the relationships between the stator voltage (*V*_*ds*_, *V*_*qs*_), stator magnetic flux (*ψ*_*m*_), and stator current (*id*_*s*_, *iq*_*s*_) may be represented as:

$$V_{ds}=r_{s}id_{s}-\omega_{e}L_{q}i_{qs}+L_{d}\frac{d_{i_{ds}}}{dt}$$ (4)

$$V_{qs}=r_{0}i_{q}s+\omega_{e}L_{d}i_{dS}+w_{e}\psi_{m}+L_{q}\frac{d_{iqs}}{dt}$$ (5)

Active power control (APC) involves deliberately regulating the actual power output from a wind turbine or a group of turbines. This is done to help balance the overall power generated on the grid with the total power consumed. So, the active and reactive power of PMSG can be determined as:

$$P_{e}=\left(\frac{3}{2}\right)[\upsilon_{q,s}i_{q,s}+\upsilon_{d,s}i_{d,s}]$$ (6)

$$Q_{e}=\left(\frac{3}{2}\right)[\upsilon_{q,s}i_{q,s}-\upsilon_{d,s}i_{d,s}]$$ (7)

$$\omega_{e}=\frac{P}{2}\omega_{g}$$ (8)

**Fig 2:**
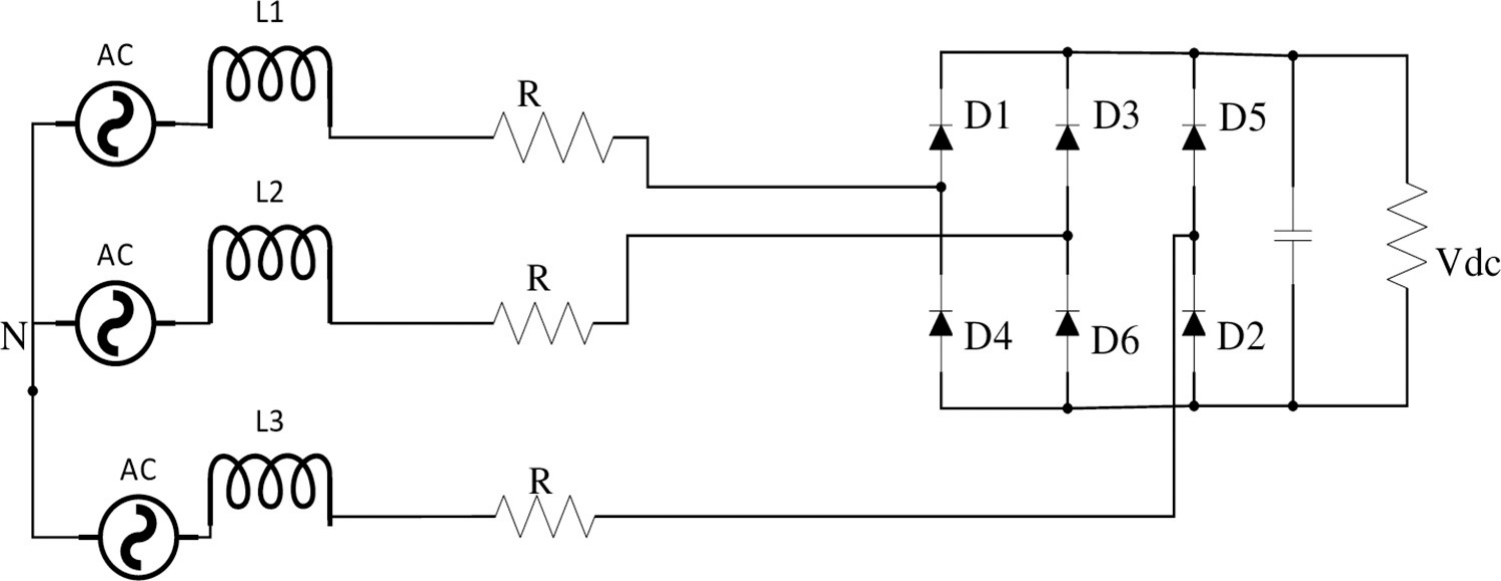
Three-phase diode bridge rectifier.


The diode rectifier is the most inexpensive and simple construction utilized in power electronic applications. It converts the output AC voltage from PMSG into DC voltage and its average output voltage. 3-ϕ Diode Bridge Rectifier shown in Fig 2, PMSG provides 3-ϕ voltage:

$$v_{rect}=\frac{3\sqrt{2}}{\pi }v_{LL}$$ (9)

Where *v*_*LL*_ represents the line-to-line voltage and *v*_*rect*_ input DC voltage for the setup boost converter.

$$e_{a}=\sqrt{2}ESin(\theta )$$ (10)

$$e_{b}=\sqrt{2}ESin(\theta -\frac{2\pi }{3})$$ (11)

$$e_{c}=\sqrt{2}ESin(\theta +\frac{2\pi }{3})$$ (12)

The current profile in CCM can be represented as:

$$i_{a}(\theta )=\frac{\sqrt{2}K}{L}[cos\varphi -cos(\theta +\varphi )]-\frac{V_{dc}}{3\omega_{L}}\theta$$ (13)

$$i_{a}(\theta )=\frac{\sqrt{2}K}{L}[cos\varphi -cos(\theta +\varphi )]-\frac{V_{dc}}{3\omega_{L}}(2\theta -\frac{\pi }{3})$$ (14)

$$i_{a}(\theta )=\frac{\sqrt{2}K}{L}[cos\varphi -cos(\theta +\varphi )]-\frac{V_{dc}}{3\omega_{L}}(\theta +\frac{\pi }{3})$$ (15)

The power of the 3- ϕ AC-DC rectifier in CCM is a function of shaft speed and capacitor voltage (steady state).


$$P_{0}=\frac{3\sqrt{2}KV_{dc}}{\pi L}Sin\theta$$ (16)

### 3.1 Operation for proposed converter

**Fig 3:**
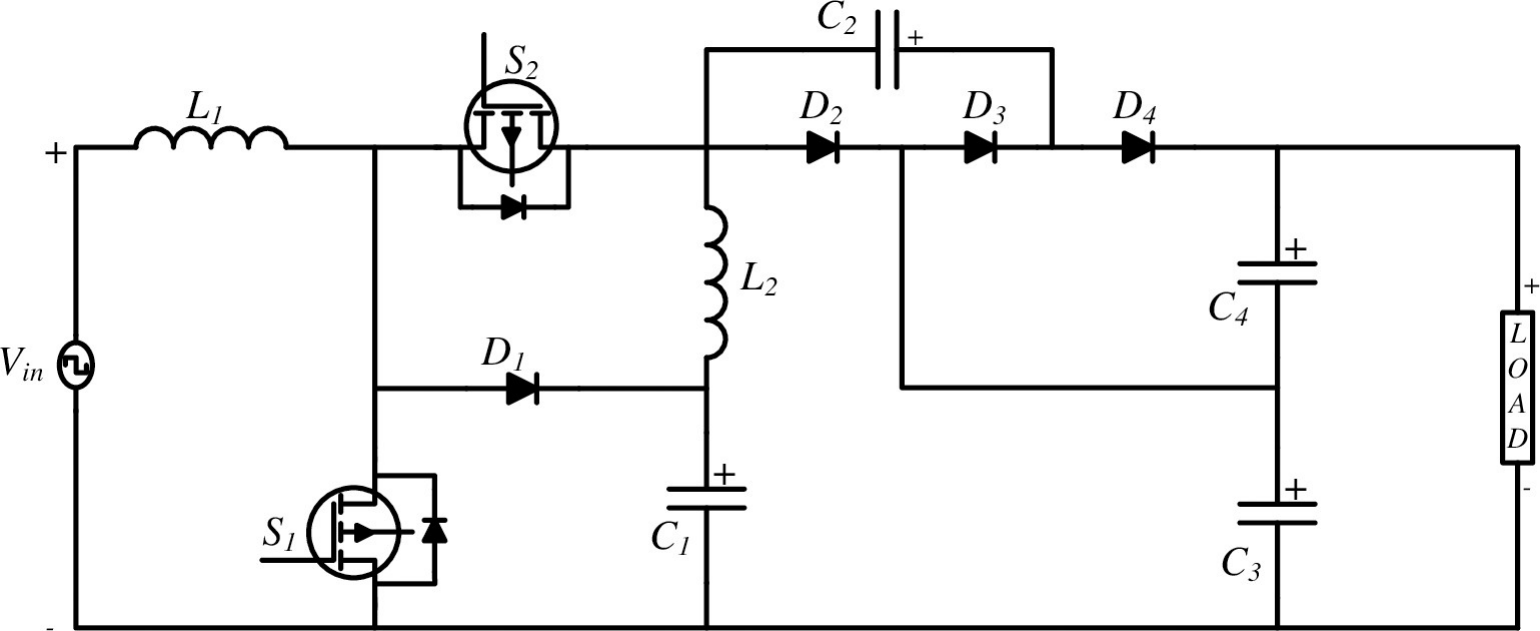
High Quadratic Step-up converter.

Fig 3 illustrates the topology of the proposed High Quadratic Step-up (HQSU) converter, which is comprised of 12 essential components, including 2 switches, 2 inductors (energy storage device), 4 capacitors (energy storage device), and 4 diodes (uncontrolled semiconductor device). Notably, all these components are considered ideal in their characteristics. The primary power source for this converter is derived from wind energy. This circuit is designed to efficiently convert wind energy into usable electrical power, making it an environmentally friendly and sustainable solution. In an ideal component scenario, each switch, inductor, capacitor, and diode operate without any inherent losses or deviations from their expected behaviour making circuit analysis more tractable. This ensures that the energy conversion process is as efficient as possible, maximizing the overall performance and reliability of the HQSU converter.

#### 3.1.1 Continuous conduction mode operation. Mode I [0 < t < DT]

**Fig 4:**
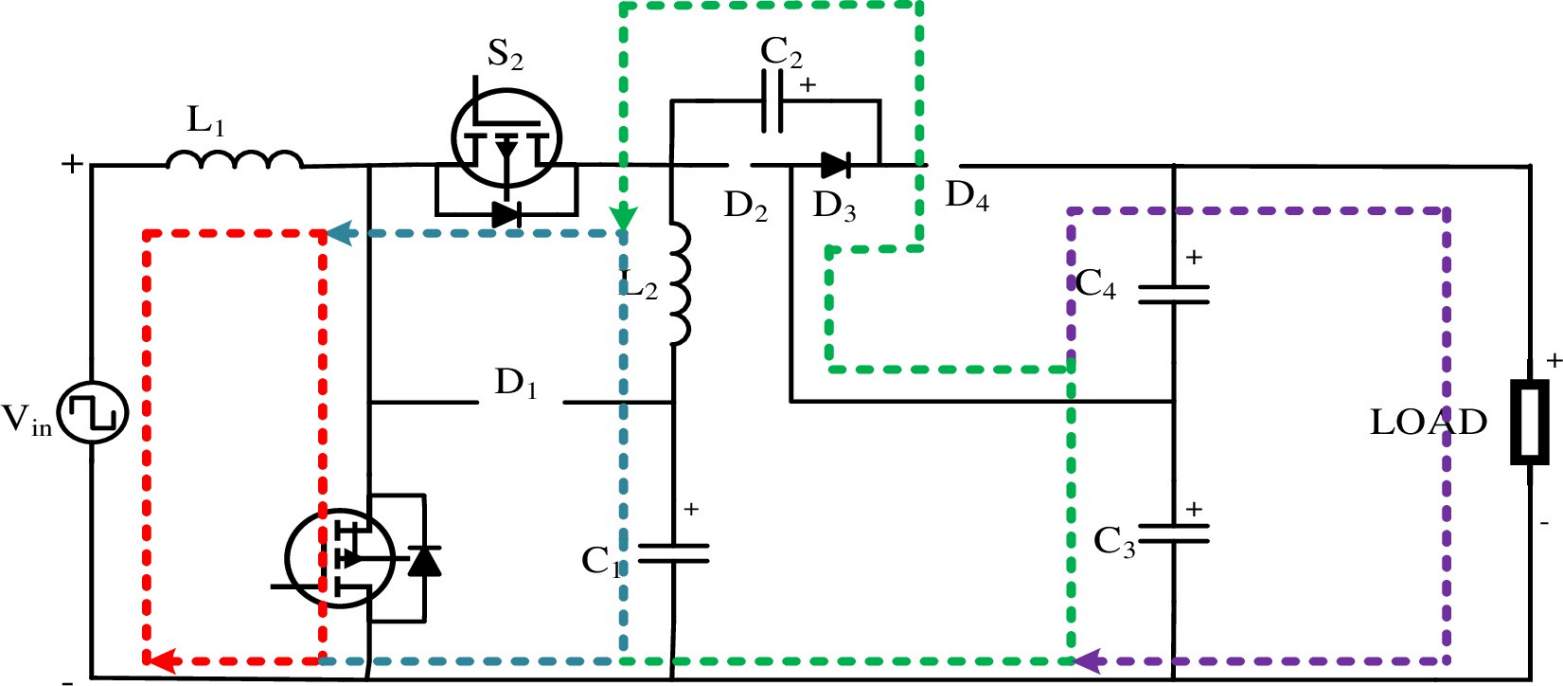
Mode 1 operation of HQSU converter.

In Mode 1, switches 1 and 2 are turned ON while diode 3 is also activated. In the initial phase, when switch S_1_ is engaged, it leads to the demagnetization of inductor (*L*_1_) with voltage (*V*_*in*_). In the subsequent step, in Loop 2, when switch (*S*_2_) is turned ON, Inductor (*L*_2_) undergoes demagnetization with Capacitor voltage (*Vc*_*in*_). Moving on to Loop 3, the Capacitor (*C*_2_) is charged by voltage (*V*_*in*_) when switch (*S*_1_) is in the ON state. Capacitor (*C*_3_) accumulates energy in the form of Capacitor voltage (*V*_*C*1_) and then discharges it through a Diode (*D*_3_). In Loop 4, the Capacitor (*C*_4_) discharges its stored energy and delivers the discharged voltage to the load side. Fig 4 shows the Mode 1 operation of the HQSU converter

$$V_{L1}=V_{in}$$ (17)

$$V_{L}=V_{C1}$$ (18)

The above two are the loop equations for continuous conduction mode at *0 < t < DT*.

Where *V*_*in*_ = Input voltage

*V*_*L*1_ = Inductor voltage (*L*_*1*_)

*V*_*C*1_ = Capacitor voltage (*C*_*1*_)

**Fig 5:**
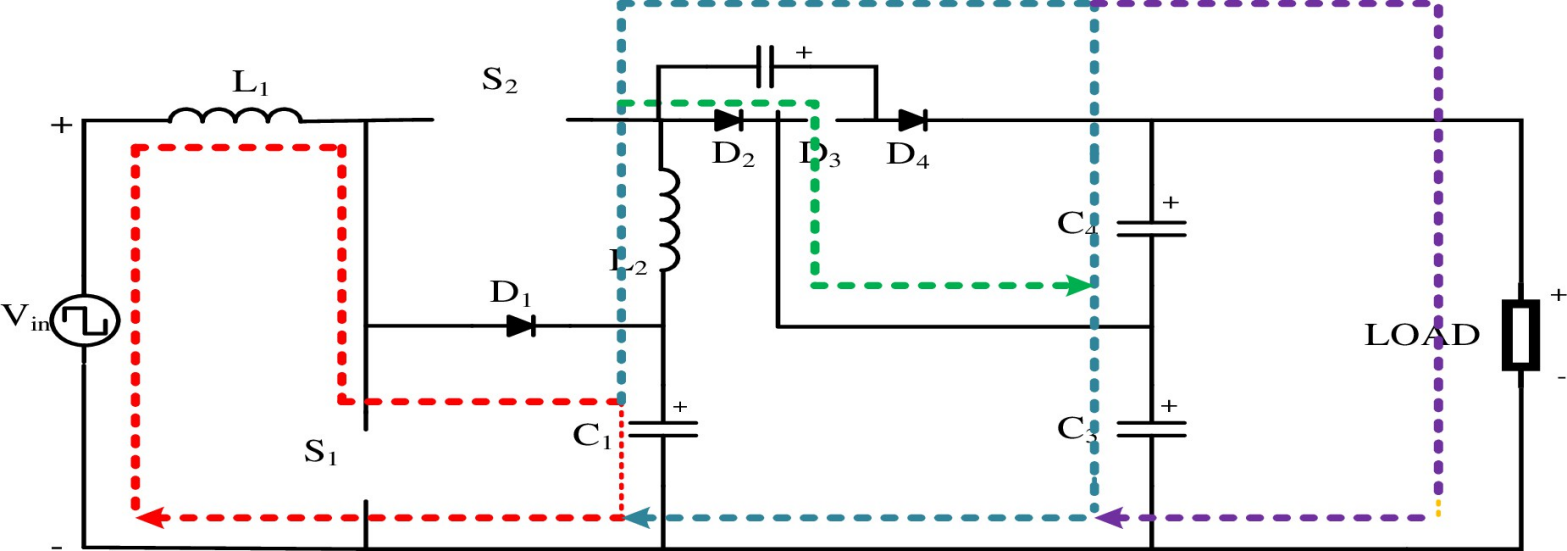
Mode 2 operation of HQSU converter.

**Mode II [DT < t < T]** The switches are off, and the capacitor *C*_1_ is charged by the inductor *L*_1_ through the diode *D*_1_. The input sources, inductors *L*_1_ and *L*_2_, are simultaneously used to charge the capacitors *C*_3_ and *C*_4_ through the respective diodes *D*_2_ and *D*_4_. Fig 5 shows the Mode 1 operation of the HQSU converter.


$$V_{L1}=V_{in}-V_{C1}$$ (19)

$$V_{L2}=V_{C1}-V_{C3}=V_{C1}+V_{C2}-V_{0}$$ (20)

The above two are the loop equations for continuous conduction mode at *DT < t < T*.

Where *V*_0_ = Output voltage

*V*_*L*2_ = Inductor voltage (*L*_*2*_)

*V*_*C*2_ = Capacitor voltage (*C*_*2*_)

#### 3.1.2 Selecting of ideal inductors

The converter’s cost and size are heavily affected by the choice of inductor, which also has a significant impact on the converter’s operational characteristics. Therefore, it is essential to meticulously design the inductor, particularly in applications such as Fuel Cell Electric Vehicles (FCEVs) where minimizing input current ripples is desirable. During the ON state of both switches in the HQSU converter, the inductors undergo magnetization, causing the inductor current to increase linearly. The inductor ripples can be evaluated by estimating the voltage across the inductors, as detailed in the Eq (21)

$$\left\{ \begin{array}{c} \Delta i_{L1}=\frac{V_{in}}{L_{1}}DTs\Rightarrow L_{1}=\frac{V_{in}D}{\Delta i_{L1}f_{s}}\\ \Delta i_{L2}=\frac{V_{C1}}{L_{2}}DTs\Rightarrow L_{2}=\frac{V_{in}D}{\Delta i_{L2}f_{s}(1-D)} \end{array}\right.$$ (21)

#### 3.1.3 Determination of capacitors

The size of the capacitor is directed by contemplations such as the maximum capacitor ripple (ΔVC), functional voltage, and the current passing through the capacitor. The equation for voltage ripple is obtained through the dynamic equations of capacitor current, and the associated formulas for capacitors are illustrated in Eq (22). In this context, a 0.1% voltage ripple is taken into account for capacitor *C*_*1*_, while capacitors *C*_*2*_, *C*_*3*_, and *C*_*4*_ are designed with a 1% voltage ripple. By employing Eq (22) and adhering to voltage ripple standards, commercially available capacitors with voltages close to the desired high values are chosen for the HQSU converter.


$$\left\{ \begin{array}{c} \Delta V_{C1}=\frac{2DI_{o}}{{C_{1}f}_{s}(1-D)}\Rightarrow C_{1}=\frac{2DP_{o}}{\Delta Vc1fsV_{0}(1-D)}\\ \Delta V_{C2}=\frac{I_{o}}{C_{2}fs}\Rightarrow C_{2}=\frac{P_{o}}{\Delta Vc2fV_{0}}\\ \Delta V_{C3}=\frac{(1+D)I_{o}}{C_{3}fs}\Rightarrow C_{3}=\frac{(1+D)P_{o}}{\Delta Vc3fsV_{0}}\\ \Delta V_{C4}=\frac{DI_{o}}{C_{4}fs}\Rightarrow C_{4}=\frac{DP_{o}}{\Delta Vc2fsV_{0}} \end{array}\right.$$ (22)

#### 3.1.4 Selection of power semiconductor switches

The choice of power semiconductor switches relies upon both the peak working voltage and the peak working current. In this scenario, the active switches are characterized by maximum operating voltages of 200V and 200V and peak operating currents of approximately 30A and 100A for the specified specifications.

## 4. Small signal analysis

Hence there are 2 modes of operation in HQSU Converter let us consider the state space equation for each mode 1 and mode 2.

MODE 1

$$\frac{di_{L1}(t)}{dt}=\frac{Lv_{in}(t)}{L_{1}}$$

$$\frac{di_{L2}(t)}{dt}=\frac{v_{C1}(t)}{L_{2}}$$

$$\frac{dv_{C1}(t)}{dt}=\frac{-iL_{2}(t)}{C_{1}}$$ (23)

$$\frac{dv_{C2}(t)}{dt}=\frac{-vC_{2}(t)}{{rC}_{2}}+\frac{vC_{3}(t)}{{rC}_{2}}$$

$$\frac{dv_{C3}(t)}{dt}=\frac{vC_{2}(t)}{{rC}_{3}}-\frac{\left(R+r)vC_{3}(t\right)}{{rRC}_{3}}-\frac{vC_{4}(t)}{{RC}_{3}}$$

$$\frac{dv_{C4}(t)}{dt}=\frac{-vC_{3}(t)}{{RC}_{4}}-\frac{vC_{4}(t)}{{RC}_{4}}$$

MODE 2

$$\frac{di_{L1}(t)}{dt}=-\frac{v_{C1}(t)}{L_{1}}+\frac{v_{in}(t)}{L_{1}}$$

$$\frac{di_{L2}(t)}{dt}=\frac{v_{C1}(t)}{L_{2}}-\frac{v_{C3}(t)}{L_{2}}$$ (24)

$$\frac{dv_{C1}(t)}{dt}=\frac{iL_{1}(t)}{C_{1}}-\frac{iL_{2}(t)}{C_{1}}$$

$$\frac{dv_{C2}(t)}{dt}=\frac{-vC_{2}(t)}{{rC}_{2}}+\frac{vC_{4}(t)}{{rC}_{2}}$$

$$\frac{dv_{C3}(t)}{dt}=\frac{iL_{2}(t)}{C_{3}}-\frac{vC_{3}(t)}{{RC}_{3}}-\frac{vC_{4}(t)}{{RC}_{3}}$$

$$\frac{dv_{C4}(t)}{dt}=\frac{vC_{2}(t)}{{rC}_{4}}-\frac{vC_{3}(t)}{{RC}_{4}}-\frac{\left(R+r)vC_{4}(t\right)}{r{RC}_{4}}$$

From the above state space equations, it is assumed that there are no energy losses in the components, thus the equivalent small-signal model is built. Below is the small signal model of mode 1.


$$\left[\begin{array}{c} \frac{di_{L1}(t)}{dt}\\ \frac{di_{L2}(t)}{dt}\\ \frac{dv_{C1}(t)}{dt}\\ \frac{dv_{C2}(t)}{dt}\\ \frac{dv_{C3}(t)}{dt}\\ \frac{dv_{C4}(t)}{dt} \end{array}\right]=\left[\begin{array}{cccccc} 0 & 0 & 0 & 0 & 0 & 0\\ 0 & 0 & \frac{1}{L_{2}} & 0 & 0 & 0\\ 0 & -\frac{1}{C_{1}} & 0 & 0 & 0 & 0\\ 0 & 0 & 0 & \frac{-1}{rC_{2}} & \frac{1}{{rC}_{2}} & 0\\ 0 & 0 & 0 & \frac{1}{rC_{3}} & -\frac{R+r}{rRC_{3}} & \frac{-1}{RC_{3}}\\ 0 & 0 & 0 & 0 & \frac{-1}{RC_{4}} & \frac{-1}{RC_{4}} \end{array}\right]\left[\begin{array}{c} i_{L1(t)}\\ i_{L2(t)}\\ V_{C1(t)}\\ V_{C2(t)}\\ V_{C3(t)}\\ V_{C4(t)} \end{array}\right]+\left[\begin{array}{c} \frac{1}{L_{1}}\\ 0\\ 0\\ 0\\ 0\\ 0 \end{array}\right]V_{in(t)}$$ (25)

The below is a small signal model of mode 2.


$$\left[\begin{array}{c} \frac{di_{L1}(t)}{dt}\\ \frac{di_{L2}(t)}{dt}\\ \frac{dv_{C1}(t)}{dt}\\ \frac{dv_{C2}(t)}{dt}\\ \frac{dv_{C3}(t)}{dt}\\ \frac{dv_{C4}(t)}{dt} \end{array}\right]=\left[\begin{array}{cccccc} 0 & 0 & \frac{-1}{L_{1}} & 0 & 0 & 0\\ 0 & 0 & \frac{1}{L_{2}} & 0 & \frac{-1}{L_{2}} & 0\\ \frac{1}{C_{1}} & \frac{-1}{C_{1}} & 0 & 0 & 0 & 0\\ 0 & 0 & 0 & \frac{-1}{rC_{2}} & 0 & \frac{1}{{rC}_{2}}\\ 0 & \frac{1}{C_{3}} & 0 & 0 & \frac{-1}{RC_{3}} & \frac{-1}{RC_{3}}\\ 0 & 0 & 0 & \frac{1}{rC_{4}} & \frac{-1}{RC_{4}} & -\frac{R+r}{rRC_{3}} \end{array}\right]\left[\begin{array}{c} i_{L1(t)}\\ i_{L2(t)}\\ V_{C1(t)}\\ V_{C2(t)}\\ V_{C3(t)}\\ V_{C4(t)} \end{array}\right]+\left[\begin{array}{c} \frac{1}{L_{1}}\\ 0\\ 0\\ 0\\ 0\\ 0 \end{array}\right]V_{in(t)}$$ (26)

Small-signal perturbations (denoted with a cap) are added to the state factors, input, and output factors. Small-signal factors are generally very low compared to their typical consistent-state values. From mode 1 and mode 2:

$$\left[\begin{array}{c} \frac{{d\hat{i}}_{L1}(t)}{dt}\\ \frac{{d\hat{i}}_{L2}(t)}{dt}\\ \frac{{d\hat{v}}_{C1}(t)}{dt}\\ \frac{{d\hat{v}}_{C2}(t)}{dt}\\ \frac{{d\hat{v}}_{C3}(t)}{dt}\\ \frac{{d\hat{v}}_{C4}(t)}{dt} \end{array}\right]=\left[\begin{array}{cccccc} 0 & 0 & \frac{-(1-d)}{L_{1}} & 0 & 0 & 0\\ 0 & 0 & \frac{1}{L_{2}} & 0 & \frac{-(1-d)}{L_{2}} & 0\\ \frac{1-d}{C_{1}} & \frac{-1}{C_{1}} & 0 & 0 & 0 & 0\\ 0 & 0 & 0 & \frac{-1}{rC_{2}} & \frac{d}{{rC}_{2}} & \frac{1-d}{{rC}_{2}}\\ 0 & \frac{1-d}{C_{3}} & 0 & \frac{d}{rC_{3}} & -\frac{Rd+r}{rRC_{3}} & \frac{-1}{RC_{3}}\\ 0 & 0 & 0 & \frac{1-d}{rC_{4}} & \frac{-1}{RC_{4}} & \frac{-R-r+Rd}{rRC_{4}} \end{array}\right]\left[\begin{array}{c} {\hat{i}}_{L1(t)}\\ {\hat{i}}_{L2(t)}\\ {\hat{v}}_{C1(t)}\\ {\hat{v}}_{C2(t)}\\ {\hat{v}}_{C3(t)}\\ {\hat{v}}_{C4(t)} \end{array}\right]+\left[\begin{array}{c} \frac{1}{L_{1}}\\ 0\\ 0\\ 0\\ 0\\ 0 \end{array}\right]{\hat{V}}_{in(t)}+\left[\begin{array}{cccccc} 0 & 0 & \frac{1}{L_{1}} & 0 & 0 & 0\\ 0 & 0 & 0 & 0 & \frac{1}{L_{2}} & 0\\ \frac{-1}{C_{1}} & 0 & 0 & 0 & 0 & 0\\ 0 & 0 & 0 & 0 & \frac{1}{rC_{2}} & \frac{-1}{{rC}_{2}}\\ 0 & \frac{-1}{C_{3}} & 0 & \frac{1}{rC_{3}} & \frac{-1}{rC_{3}} & 0\\ 0 & 0 & 0 & \frac{-1}{rC_{4}} & 0 & \frac{1}{rC_{4}} \end{array}\right]\left[\begin{array}{c} I_{L1}\\ I_{L2}\\ V_{C1}\\ V_{C2}\\ V_{C3}\\ V_{C4} \end{array}\right]\hat{d}$$ (27)

In general, the input voltage *Vin*, the output voltage *V*_*0*_, the load resistance *R*, the duty ratio (*D*), and the inductor currents *i*_*L1*_*(t)* and *i*_*L2*_*(t)* and capacitor voltages *V*_*C1*_*(t)*_,_ *V*_*C2*_*(t)*_,_ *V*_*C3*_*(t)* and *V*_*C4*_*(t)* are regarded as the state variables system input that is under control.

$\frac{di_{L1}(t)}{dt}$ = Rate of change of inductor current (*L*_1_) with respect to time

$\frac{di_{L1}(t)}{dt}$ = Rate of change of inductor current (*L*_2_) with respect to time

$\frac{dv_{C1}(t)}{dt}$ = Rate of change of capacitor voltage (*v*_*C*1_) with respect to time

$\frac{dv_{C2}(t)}{dt}$ = Rate of change of capacitor voltage (*v*_*C*2_) with respect to time

$\frac{dv_{C3}(t)}{dt}$ = Rate of change of capacitor voltage (*v*_*C*3_) with respect to time

$\frac{dv_{C4}(t)}{dt}$ = Rate of change of capacitor voltage (*v*_*C*4_) with respect to time

## 5. Performance comparison

**Fig 6:**
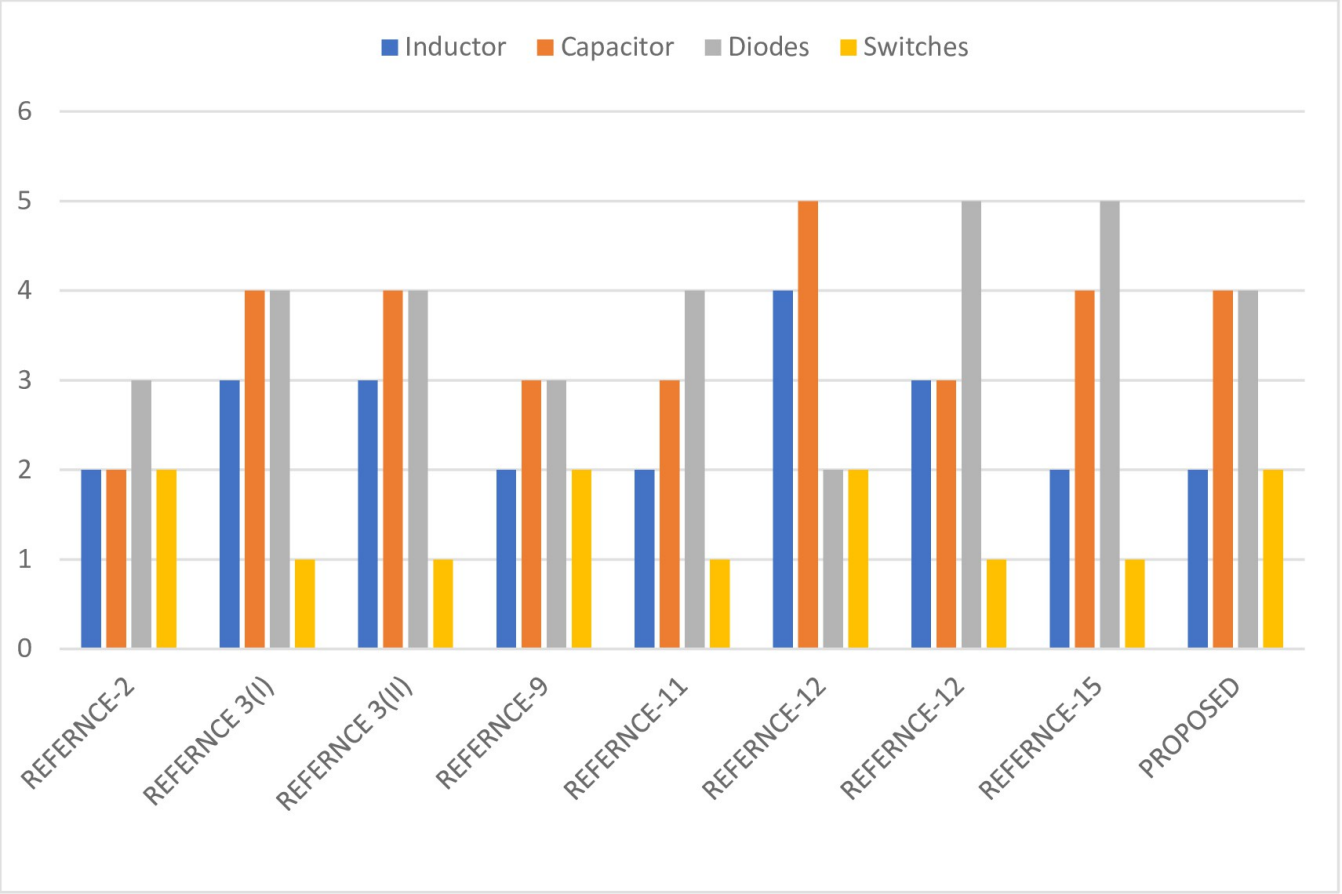
Comparison of required numbers of components for DC-DC converters.

The above bar graph shown in Fig 6 summarises various DC-DC converters documented in a literature review. While these converters share a common underlying principle, what distinguishes the proposed DC-DC converter is its unique characteristic: it maintains a consistent current ripple and doubles the output voltage compared to other converters. Furthermore, the proposed converter boasts a relatively lower level of complexity than its counterparts. A comparison is made based on the quantities of inductors, diodes, switches, and capacitors employed in the system.

**Inductor:** Comparing the component values, [35] and [26] (both Type 1 and Type 2) have the most inductors, while the proposed converter has the fewest at 2. This suggests that the compared converter might experience increased size, cost, and losses due to the excess inductors (Here the Inductors are indicated using the colour blue).

**Capacitor:** In terms of capacitors, [35] has the highest value of 5, while [25] and [32] have the lowest at 2. The proposed converter contains 4 capacitors, resulting in a smoother output and improved filtering (Here the capacitors are indicated using the colour orange).

**Diode:** With diodes, [36] and [38] have the highest value of 5, whereas [25] has the lowest with 2 diodes. The proposed converter has 4 diodes, leading to smoother operation and enhanced filtering, though this might increase size and cost (Here the diodes indicate the use of the colour ash).

**Switch:** [35] has the most switches with a value of 2, while [34, 36, 38], and [32] all have the lowest value of 1. The proposed converter has 2 switches, potentially causing some inefficiency compared to the presence of high switch counts in other converters (Here the Switches are indicated using the colour yellow).

## 6. Results and discussion

**Fig 7:**
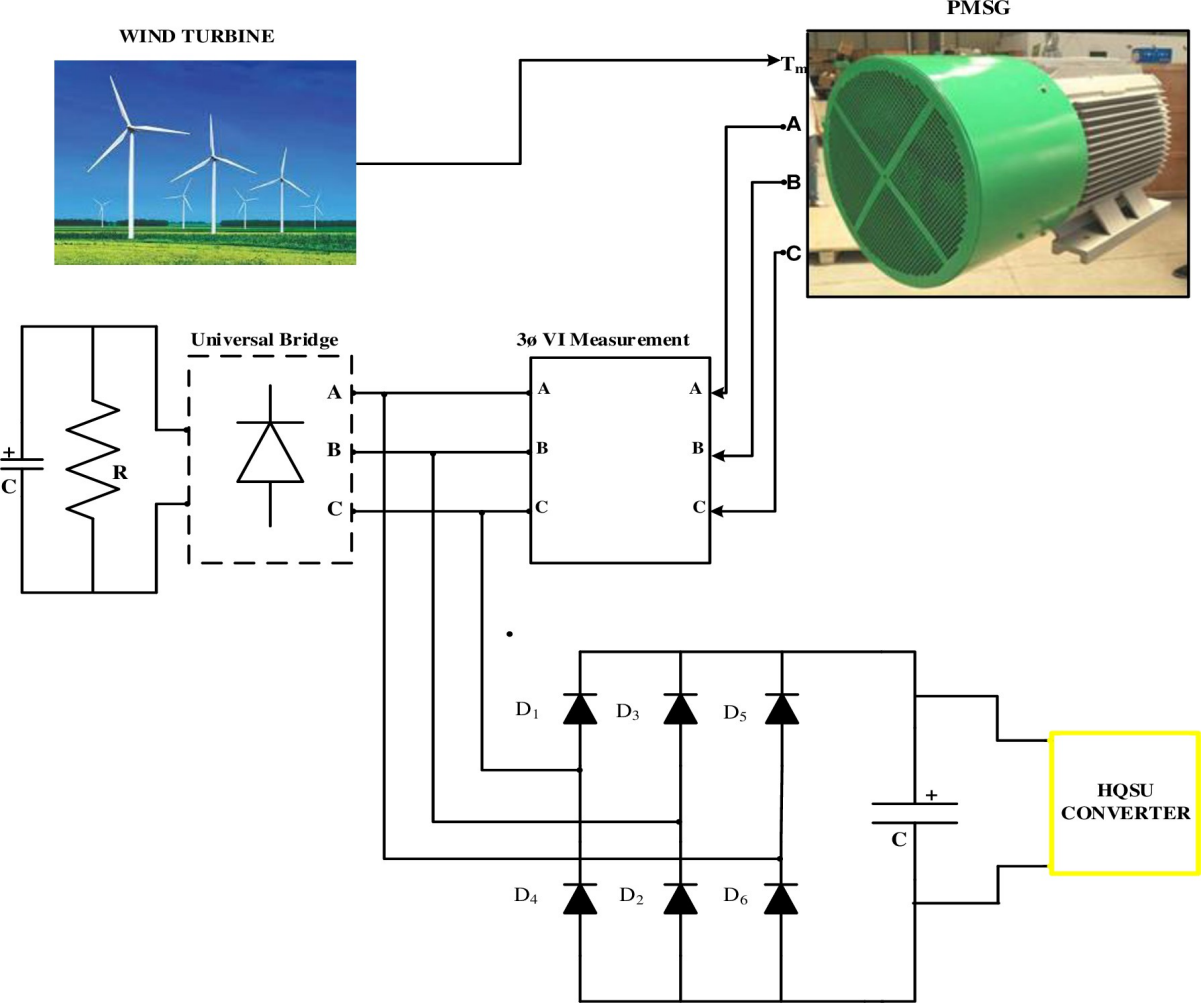
Proposed block diagram of PMSG based with HQSU.

The Fig 7 shows the block diagram of a wind turbine model with the Proposed PMSG based with HQSU. The model incorporates elements like a wind turbine block, a PMSG, and a three-phase diode bridge rectifier. During the testing phase, the model evaluates a range of wind speeds, varying from 8 m/s to 12 m/s, while the mechanical output power remains constant at 6 kW. The PMSG parameters include 8 poles, a stator resistance of 0.425 ohms, and a stator inductance of 8.35 mH. Table 1 presents the simulation values of the Wind model with PMSG connected.

**Table 1:** Parameters of WPCS with PMSG connected.

**Turbine Parameters**	Wind speed	16kW
	Mechanical output power	2m/s
**PMSG Parameters**	Number of poles	8
	Stator Resistance	0.425ohm
	Stator Inductance	8.35mH

Each component values of the proposed HQSU (Fig 3) are designed based on the analytical equations. The values of all the elements are presented below in Table 2.

**Table 2:** Parameters of High Quadratic Step-up converter.

PARAMETERS	SPECIFICATIONS
Input voltage	100V
Output voltage	750V
Switching frequency	50 kHz
Inductors	
*L*_1_	193.50*10^-3^H
*L*_2_	374.818*10^-3^H
Capacitors	
*C*_1_	49.975*10^-6^F
*C*_2_	26.6666*10^-6^F
*C*_3_	25.8*10^-6^F
*C*_4_	39.566*10^-6^F
Load (*R*_*L*_*)*	112.5Ω
Duty ratio	0.48375
Output power	5 kW

### 6.1 Closed loop control scheme

**Fig 8:**
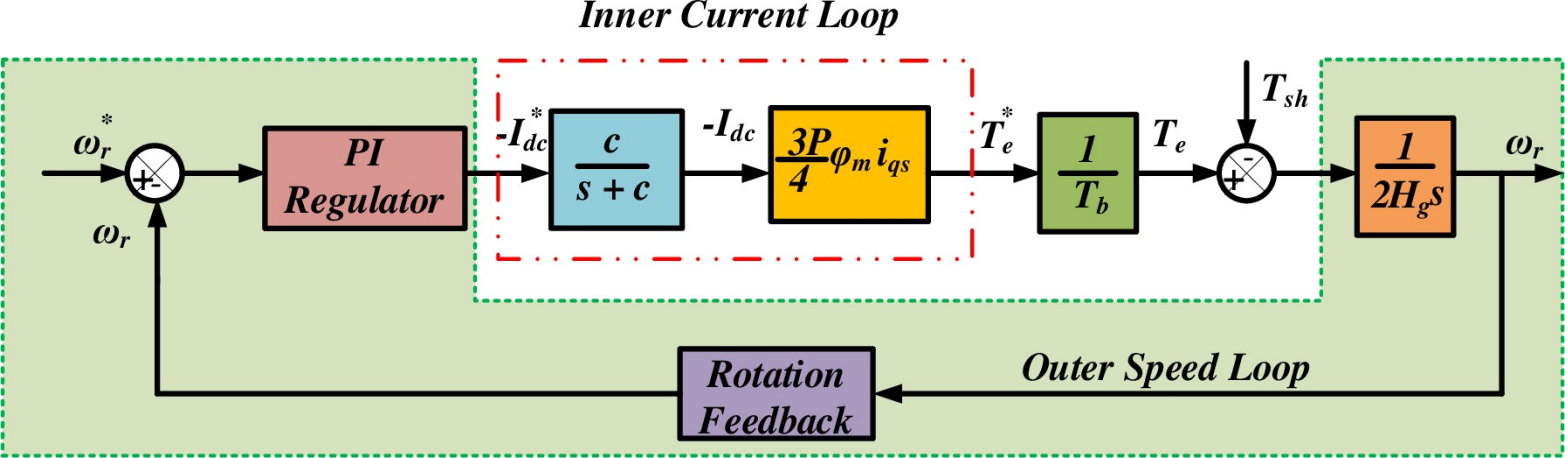
Closed loop control using double loop scheme (inner current & outer speed).

The Fig 8 illustrates the schematic structure of the double loop control system, with the speed controller loop serving as an outer loop and the current control loop as the inner loop, as explained in reference [45]. The speed sensor captures the actual rotor speed, denoted as *ω*_*r*_, in the speed control loop. The speed error is determined by subtracting the desired rotor speed $\omega_{r}^{*}$ from the actual rotor speed *ω*_*r*_. The speed control loop utilizes a proportional-integral (PI) regulator. The reference current $i_{qs}^{*}$ is determined from the speed control loop and subtracted from the input inductor current of HQSU converter *i*_*L*1_ to determine the error in the inner current control loop.

The controller supplies the desired torque $T_{e}^{*}$ and the desired q-axis stator current $i_{qs}^{*}$ for the current control loop. HCC is employed to calculate the control signal due to the minimal inertia of the current control loop. Within the context of HCC, the current *i*_*L*1_ is constrained to a specific range defined by the tolerance band of the $i_{qs}^{*}$. The frequency of the switching waveform is constrained by the careful choice of the hysteresis band (HB).

**Fig 9:**
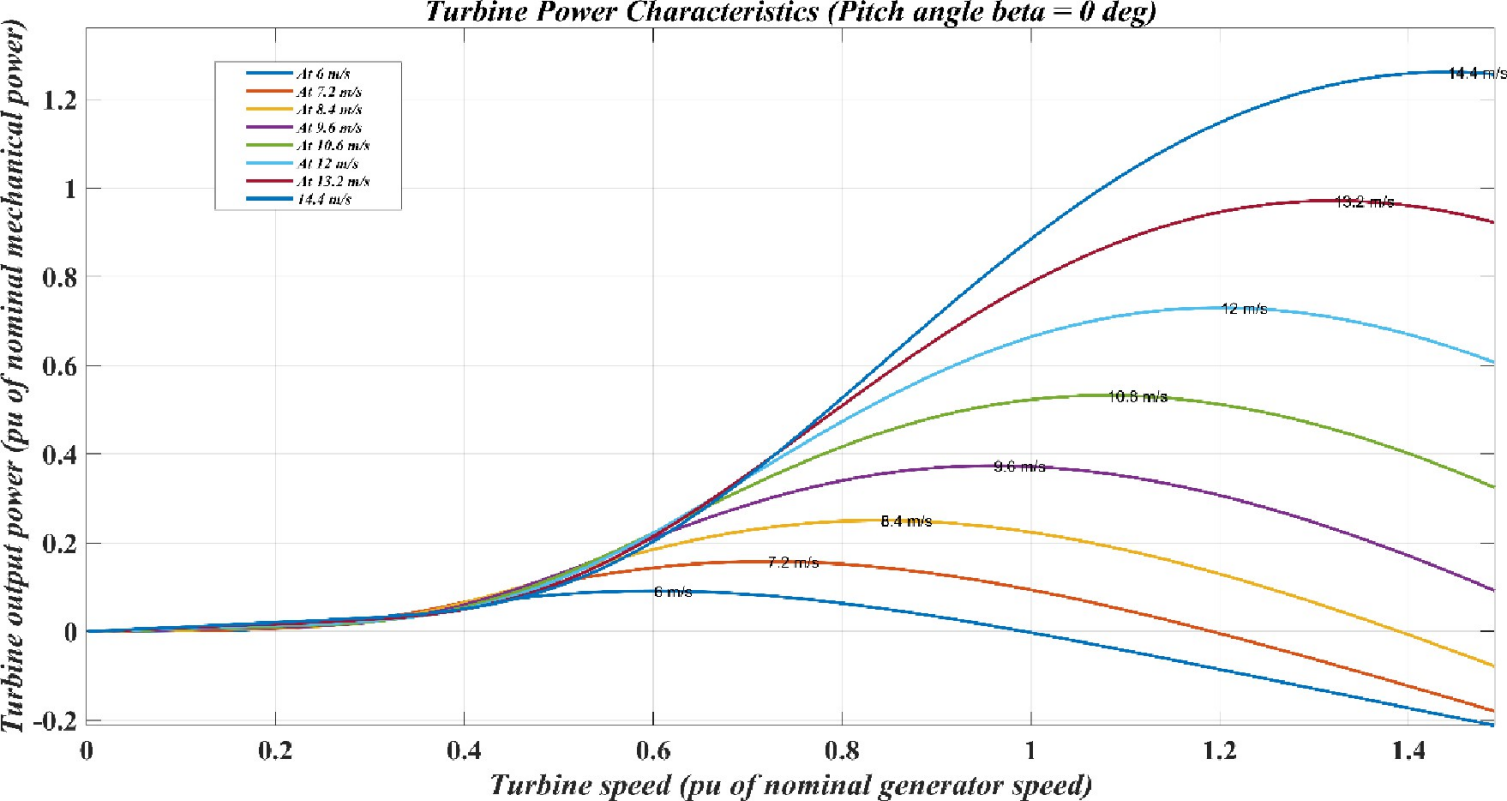
Characteristics of wind turbine.

Fig 9 shows the characteristics of the wind turbine plotted between turbine output power and turbine speed. It displays the different levels of speed of the wind turbine. The curve aids in energy assessment, warranty formulations, and performance monitoring of the turbines.

**Fig 10:**
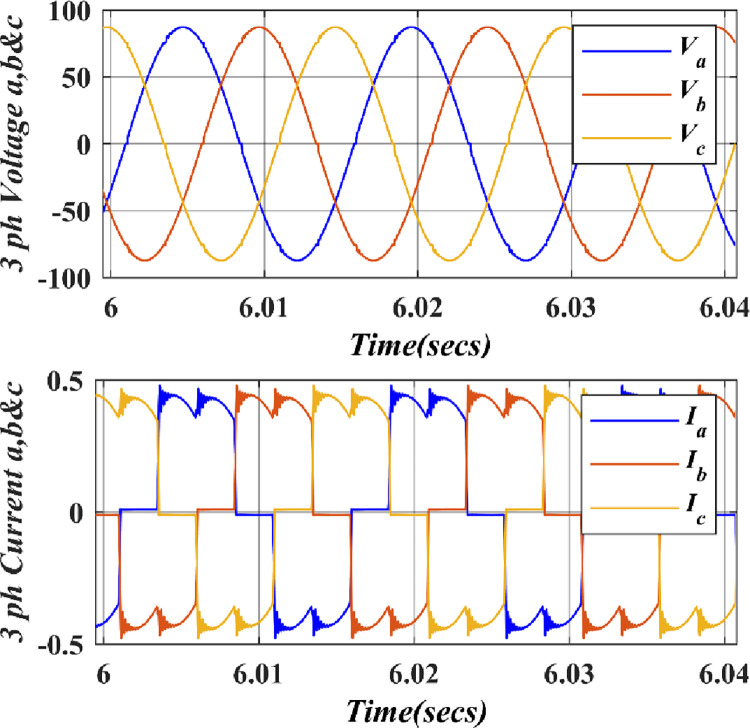
Three–phase simulated waveforms of voltages and currents of PMSG.

In Fig 10, the graph illustrates the three–phase waveforms of voltages and currents of a PMSG as they vary with changing wind speed. This variation in wind speed leads to shifts in both the amplitude (voltage level) and frequency (the rate of oscillation) of the PMSG’s output voltage. This graph is a valuable representation of the generator’s behavior in response to varying environmental conditions.

**Fig 11:**
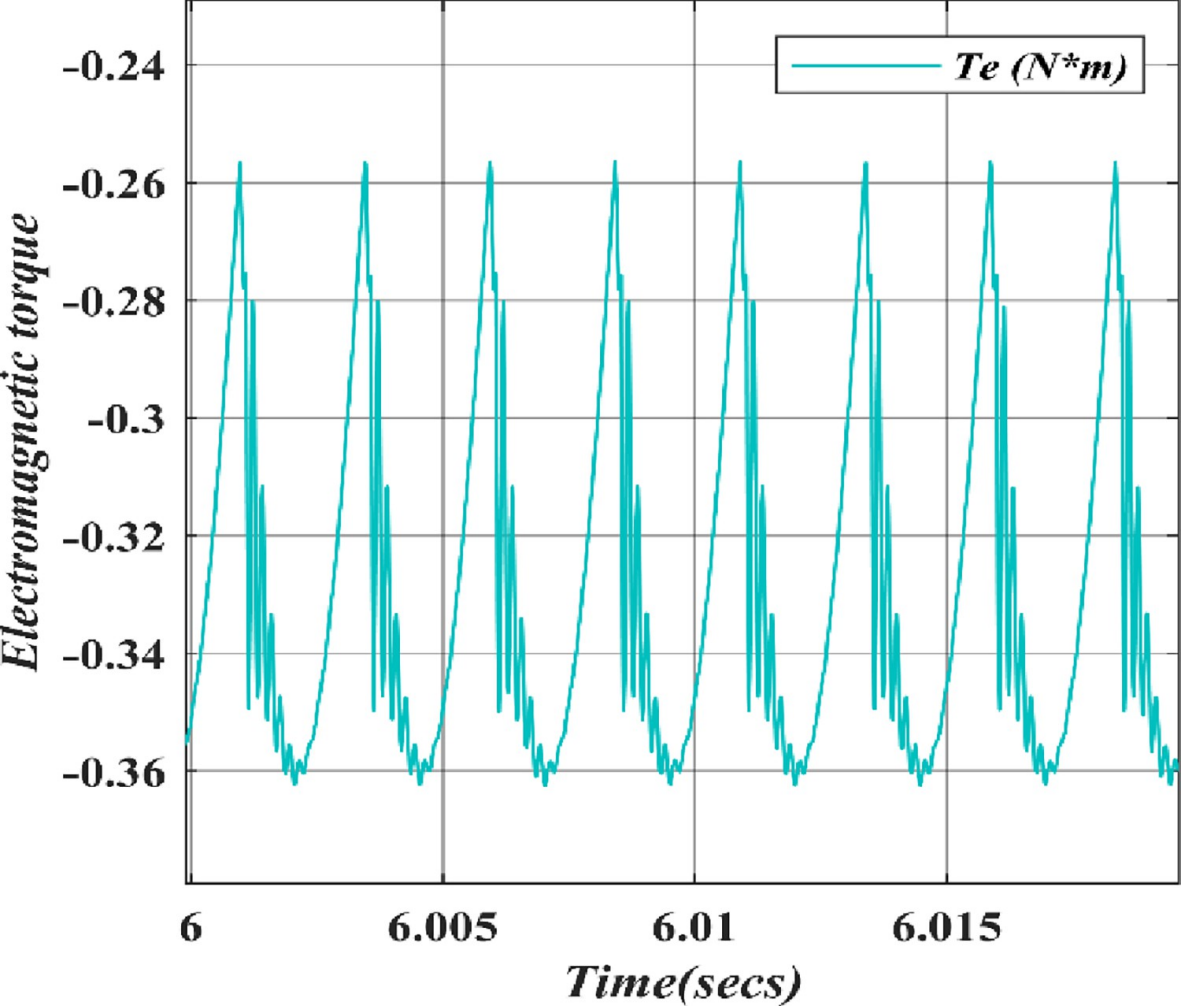
Electromagnetic torque of wind turbine.

**Fig 12:**
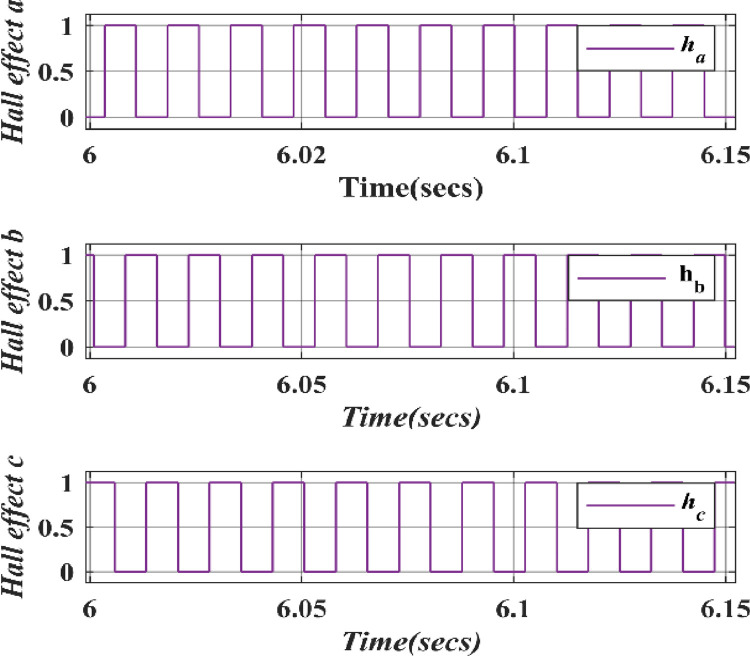
Hall effect of wind speed.

**Fig 13:**
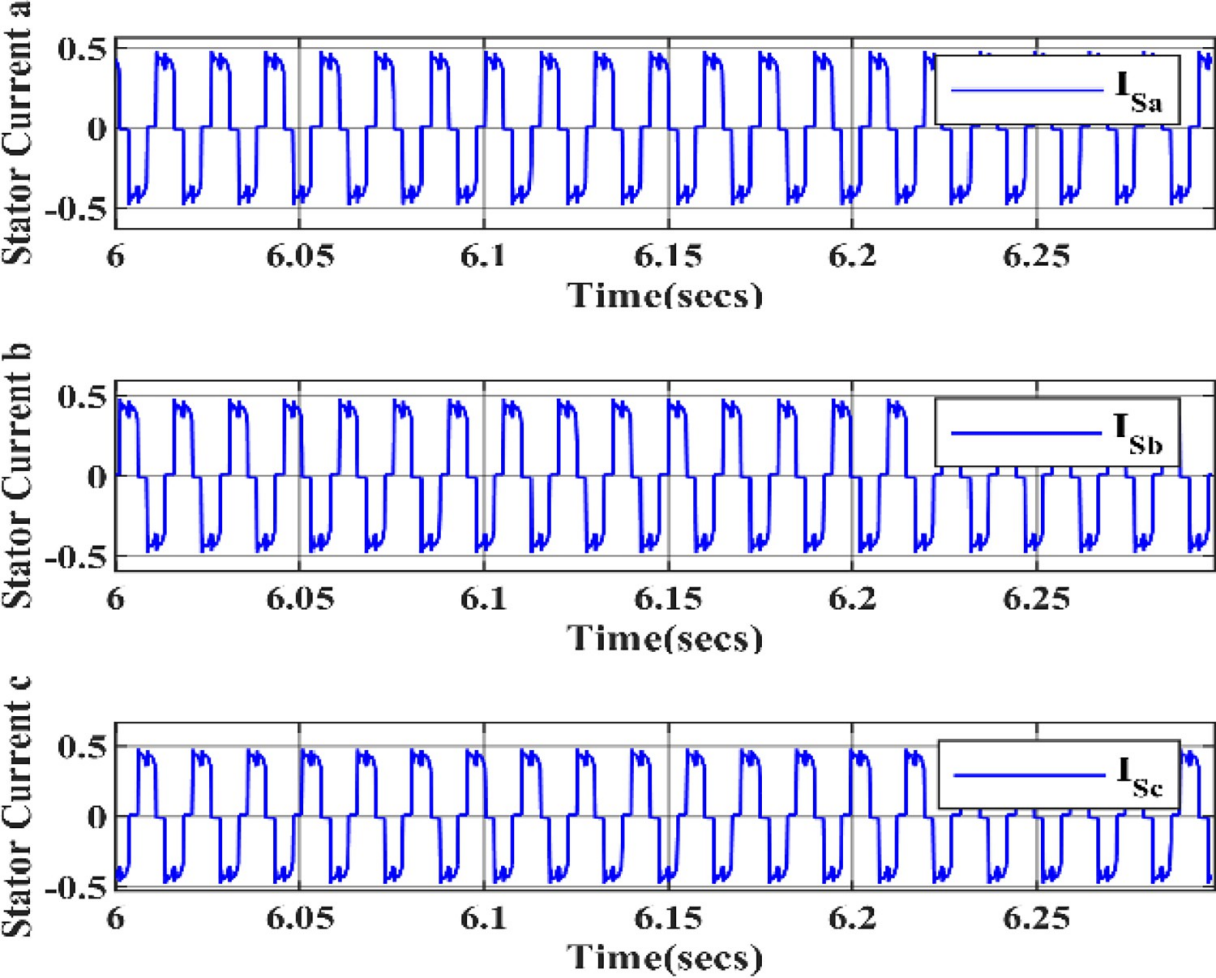
Stator currents of wind turbine.

In Figs 11–13 the graph shows the Electromagnetic torque, Hall Effect and Stator currents, respectively. Initially, the Rotor speed starts at 0, increasing to 100, and then becomes constant, which stabilizes the wind turbine system as it responds to changes in wind speed and ultimately reaches a steady-state operation where the rotor speed remains constant, allowing for efficient power generation. The electromagnetic torque initially drops to zero, then becomes negative, and finally stabilizes when a wind turbine is linked to a PMSG and a HQSU converter. Stator current varies with wind speed, exhibiting fluctuations and sine-wave-like patterns due to converter operation.

**Fig 14:**
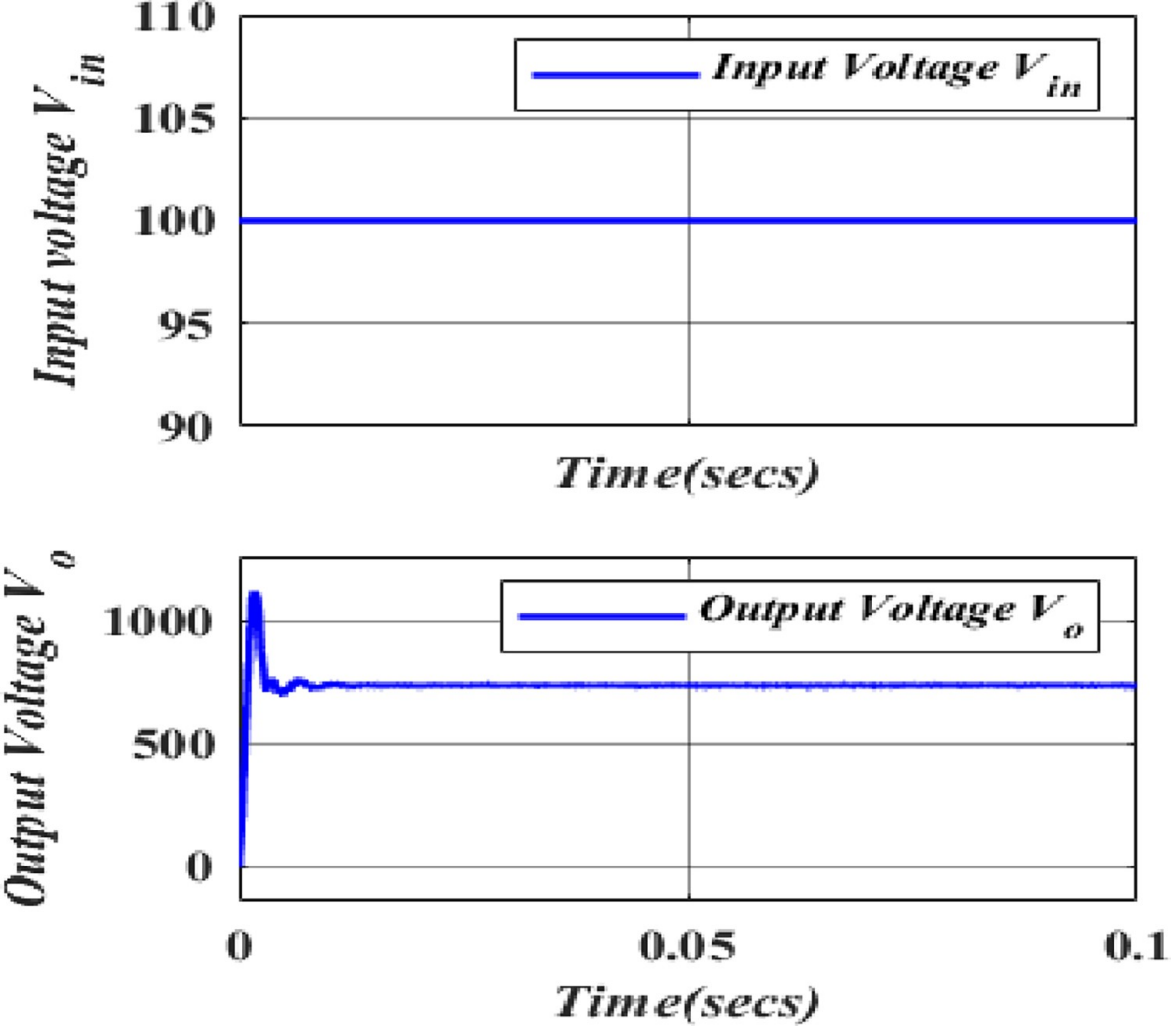
Input and output voltage waveform of High Quadratic step-up converter.

**Fig 15:**
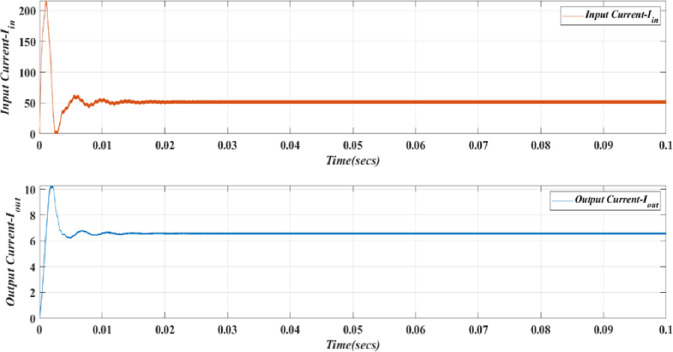
Input and output current waveform of High Quadratic step-up converter.

Fig 14 illustrates the input and output voltages and their correlation in the proposed converter. Operating at a frequency of 50Hz with a duty cycle of 0.48375 (48.375%). The constant output average voltage of 738.8V has achieved with simulated model due to the presence of internal resistances but as per theoretical, it gives output voltage as 750V. The deviation is less than 1.5%, which shows the validity of the proposed converter. Hence, this converter consistently boosts the output voltage, ensuring a reliable performance as shown in Fig 15.

**Fig 16:**
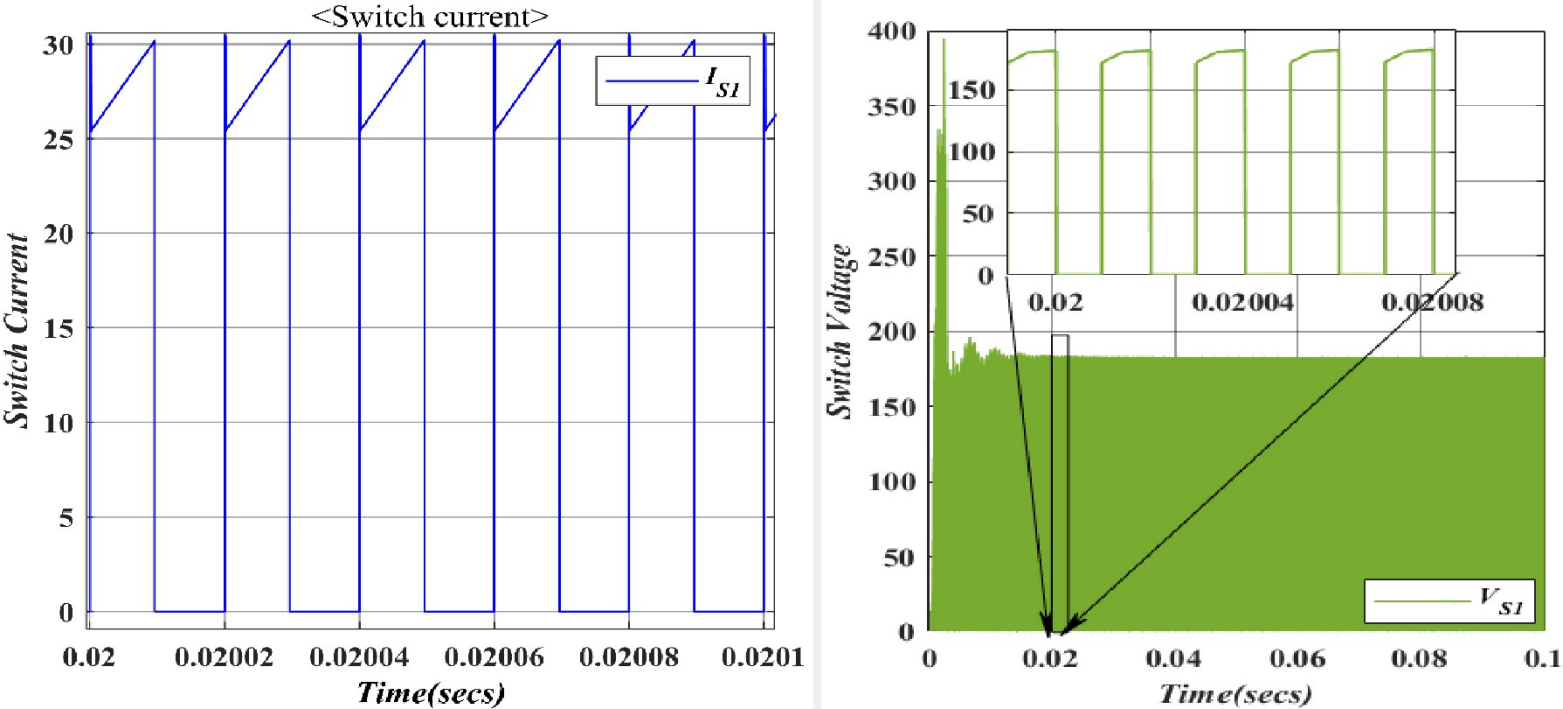
Waveform of voltage and current for switches (*S*_1_).

**Fig 17:**
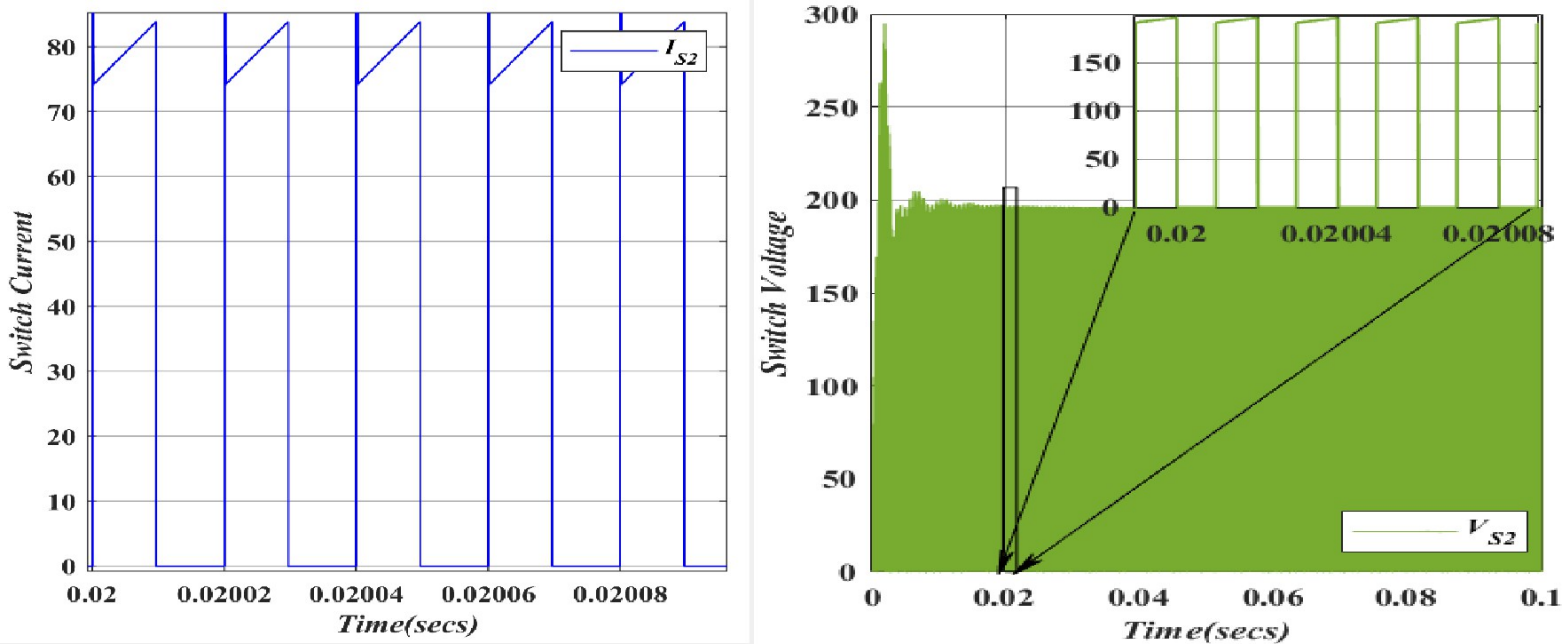
Waveform of voltage and current for switches (*S*_2_).

With the switch (*S*_1_) demagnetization of the inductor (*L*_1_) takes place. The switch voltage is close to the input voltage, allowing current to flow from the input to the associated capacitor. The voltage across the switch is typically low, near the input voltage. Current flows from the input source through the switch to charge the associated capacitor. This results in a positive current flowing through the switch. With the switch (*S*_2_) demagnetization of the inductor (*L*_2_) takes place. Figs. 16, 17 depicts the voltages and currents of (*S*_1_, *S*_2_). For *S*_*1*_ the Voltage registers at 182.6V, accompanied by a current of 25A. Meanwhile, *S*_*2*_ exhibits a voltage of 196.2V and a current of 84A. The blue colour indicates the Voltage and current of *S*_*1*_, whereas the orange colour demonstrates the Voltage and current of *S*_*2*_ as a result the theoretical and simulation value have shown the same.

Upon the initial engagement of switch *S*_*1*_, the resulting demagnetization of the inductor (*L*_*1*_) is observed with a voltage input (*V*_*in*_ = 100V). As inductors, being passive electronic components, resist sudden changes in current, they may exhibit characteristics that appear "unstable" during the transition state. Examining the voltage and current patterns in the inductor (*L*_*1*_) reveals that rapid changes in current can lead to voltage spikes across the inductor due to the back electromotive force (back EMF) generated. Furthermore, the switch’s impact influences the transient behavior (*S*_*1*_). In a steady-state condition, the graph indicates a stable output.

**Fig 18:**
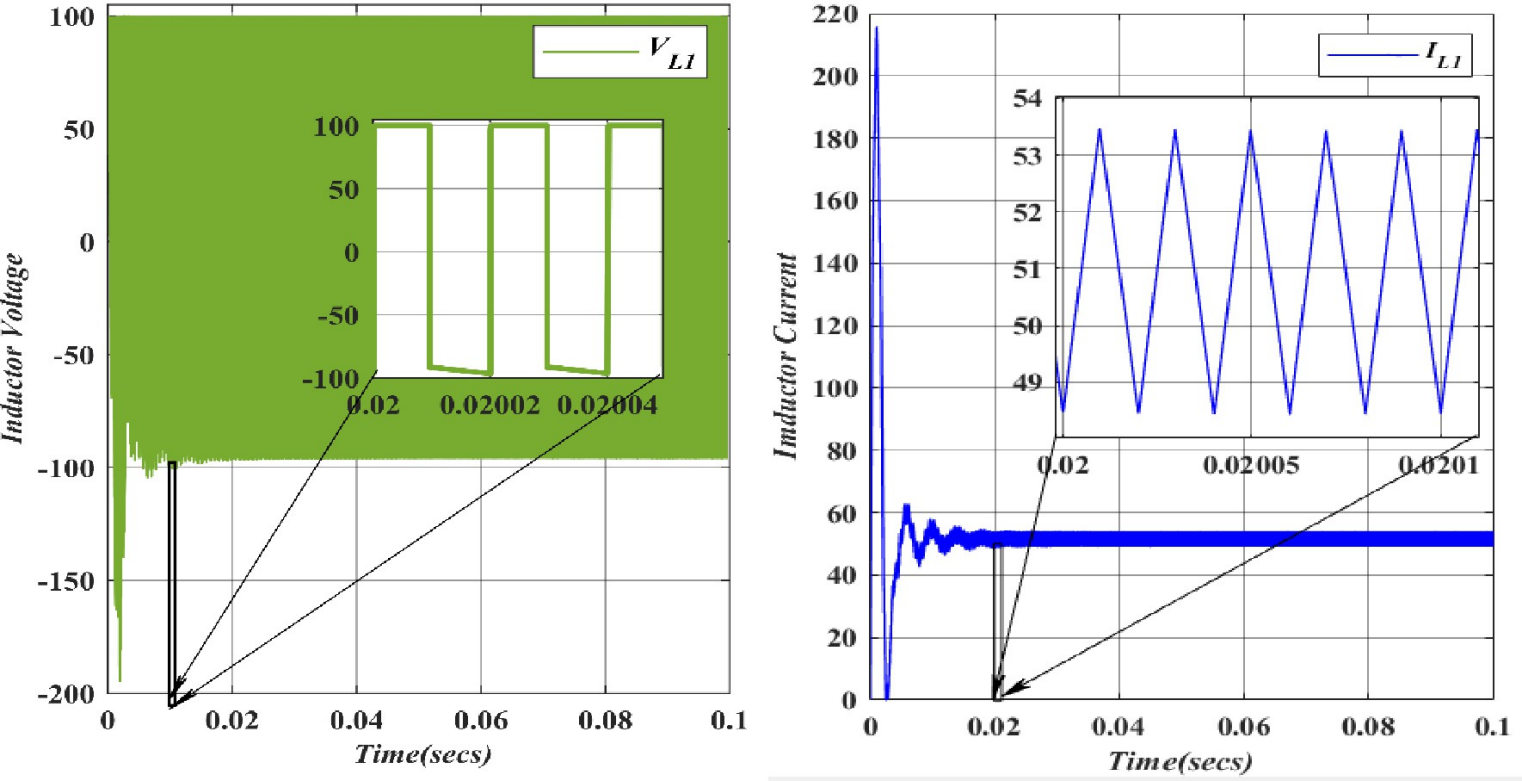
Waveforms of inductor (*L*_1_) voltage and current.

**Fig 19:**
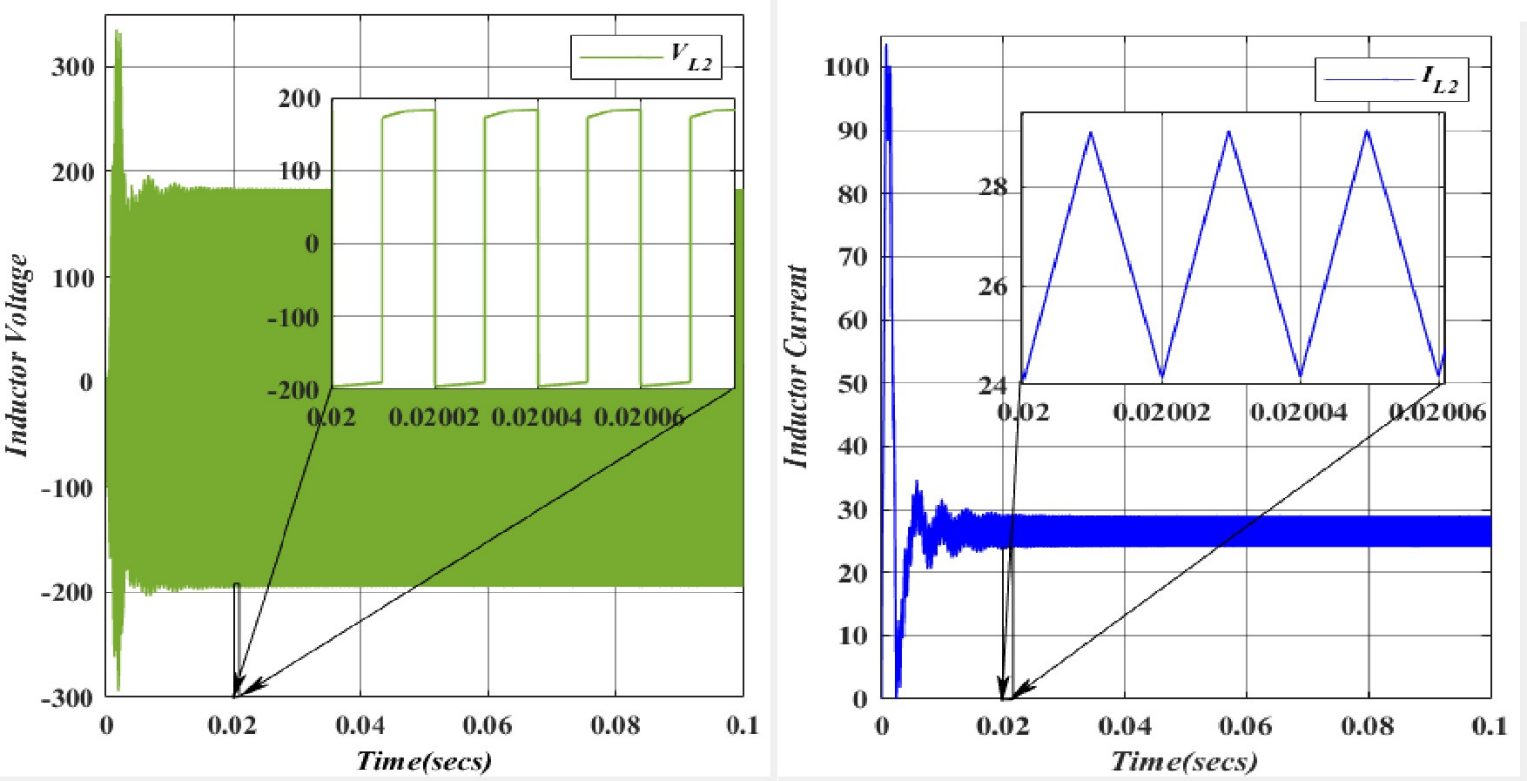
Waveforms of inductor (*L*_2_) voltage and current.

Upon activating switch *S*_*2*_, the demagnetization of the Inductor (*L*_*2*_ = 374.818*10^-3^H) is described with Capacitor voltage (*C*_*1*_ = 49.975*10^-6^F). When subjected to abrupt changes in current, inductors generate a back electromotive force opposing the change and may experience magnetic saturation, resulting in inefficient storage of magnetic energy. This behaviour can lead to unpredictable outcomes and negative voltage or current waveforms. Figs 18 and 19 depict the voltage and current characteristics for (*L*_*1*_, *L*_*2*_). For *L*_*1*_, the voltage is 100V, and the current is 51.5A.

In contrast, *L*_*2*_ exhibits a voltage of 183.3V and a current of 26.5A. Both *L*_*1*_ and *L*_*2*_ display a ripple current of 5A. The blue colour represents the Voltage and current of *L*_*1*_, while the orange colour represents the Voltage and current of *L*_*2*_. A comparison of these values with the theoretical and simulation data confirms their alignment.

The charging of the Capacitor (*C*_*1*_ = 49.975*10^-6^F) is facilitated by the Inductor (*L*_*1*_) through the Diode (*D*_*1*_). In the forward-biased state of the diode, its voltage remains relatively constant and closely tracks the capacitor voltage. Consequently, a positive current flow through the diode, charging Capacitor (*C*_*3*_) during this phase. The capacitor (*C*_*2*_ = 26.6666*10^-6^F) undergoes charging from Voltage (*V*_*in*_ = 100V) upon the activation of Switch (*S*_*1*_). It is important to note that capacitors resist abrupt changes in voltage. The voltage and current waveforms of Capacitor (*C*_*2*_) demonstrate that during the initial charging or discharging, voltage and current can experience rapid changes, leading to transient effects and potential voltage spikes across capacitors. The capacitor voltage (*C*_3_ = 25.8*10^-6^F) is charged from the Capacitor voltage (*C*_1_ = 26.6666*10^-6^F) and discharged through the diode (*D*_3_). However, the Capacitor (*C*_3_) will not be able to exceed this voltage since the diode (*D*_3_) will be reverse-biased and block any current from flowing back into the capacitor (*C*_1_). In this scenario, diode (*D*_3_) allows current to flow out of the capacitor (*C*_3_) while blocking any reverse current from the capacitor (*C*_3_) back into the capacitor (*C*_1_).

**Fig 20:**
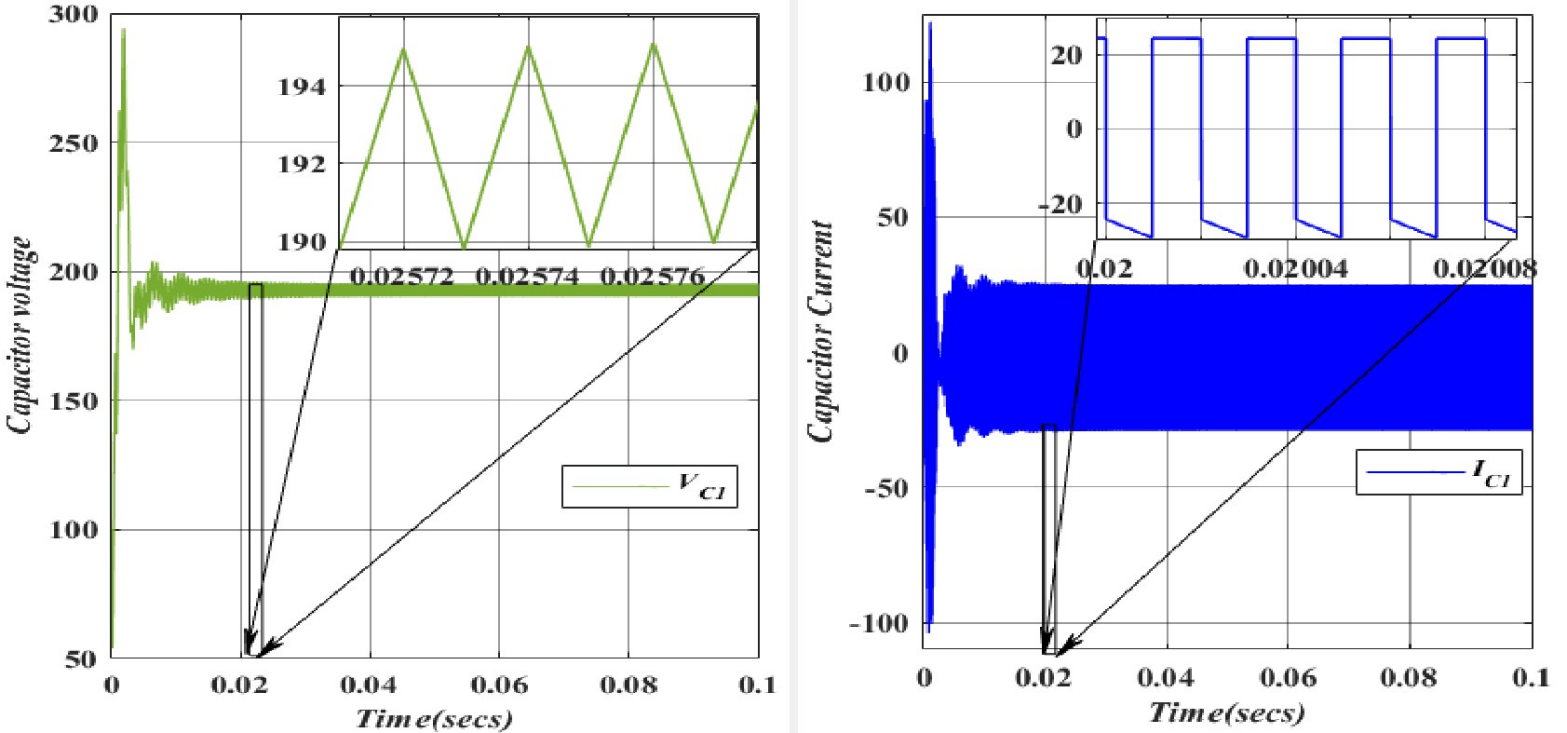
Waveforms of capacitor (*C*_1_) voltage and current.

**Fig 21:**
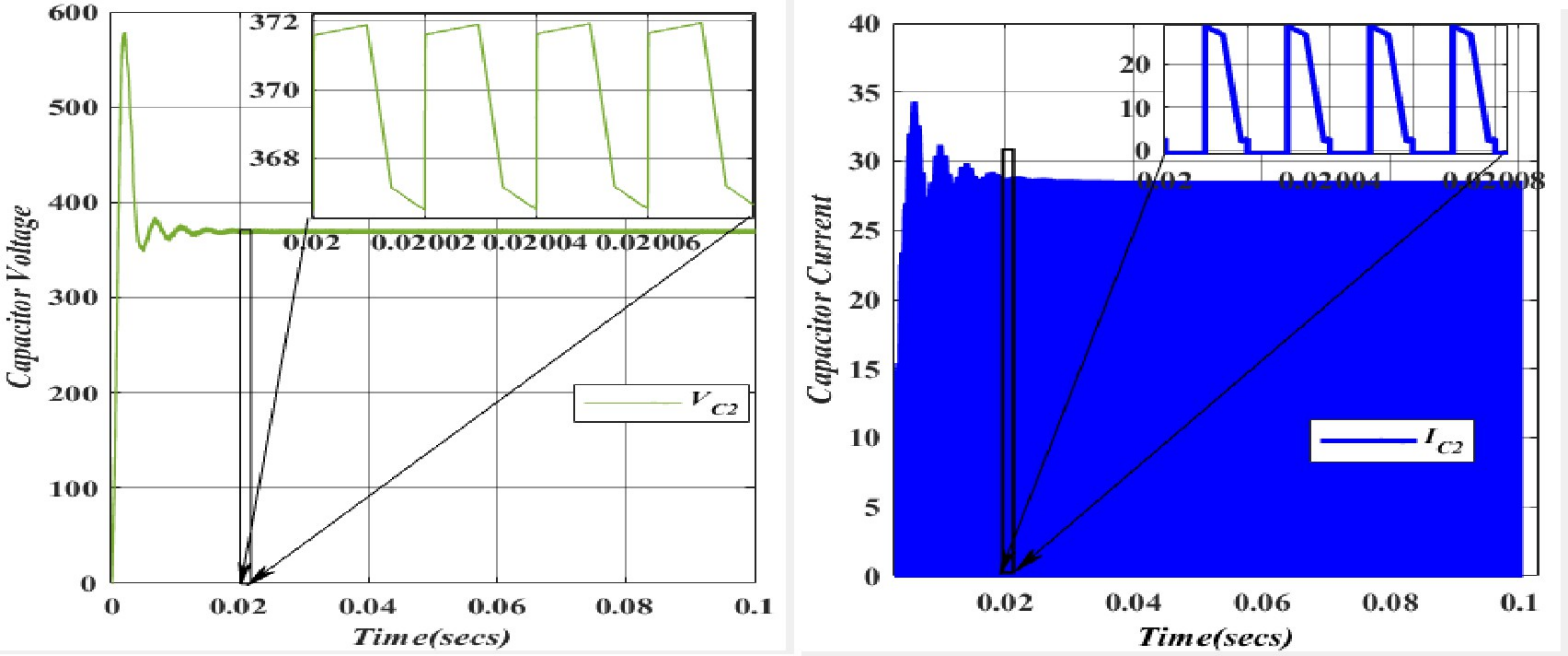
Waveforms of capacitor (*C*_2_) voltage and current.

**Fig 22:**
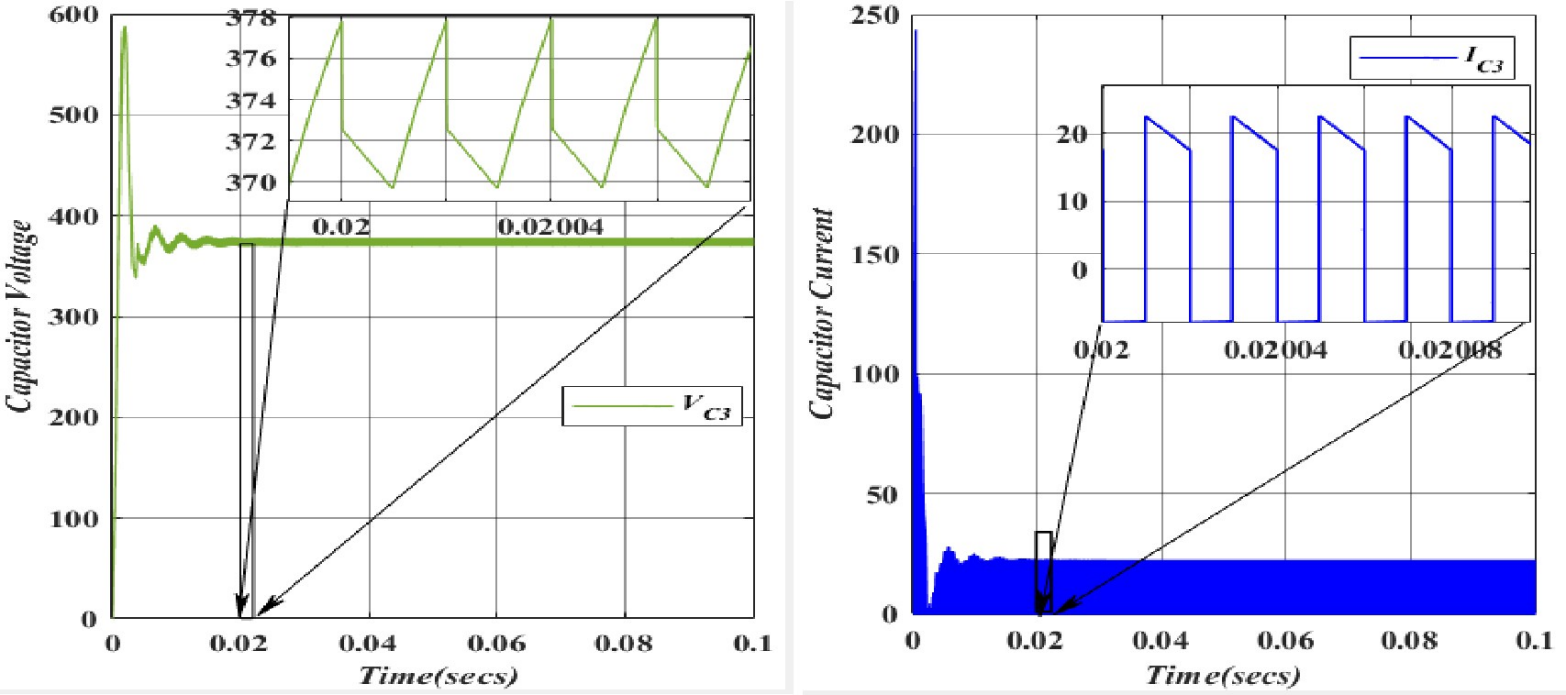
Waveforms of capacitor (*C*_3_) voltage and current.

**Fig 23:**
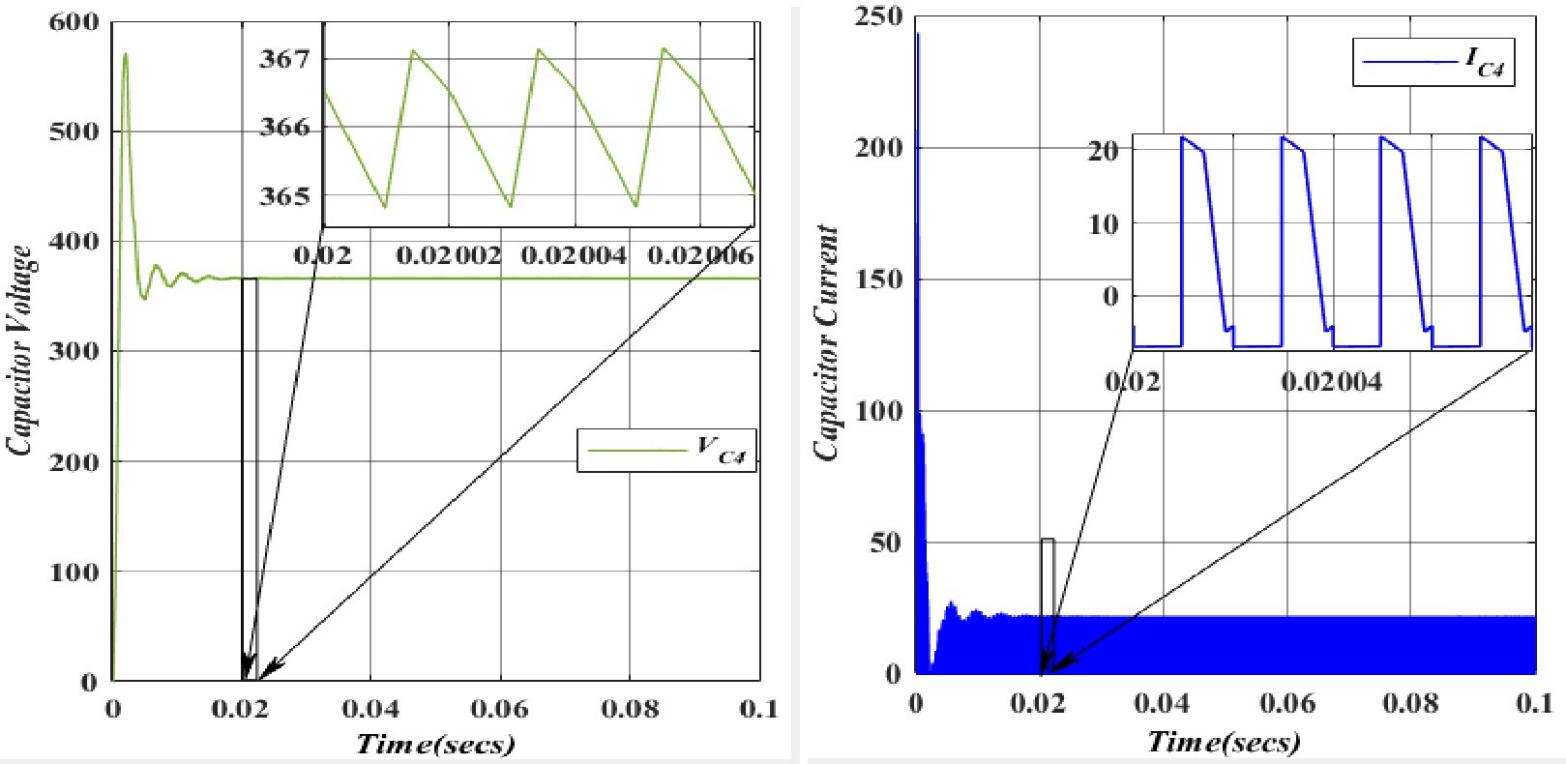
Waveforms of capacitor (*C*_4_) voltage and current.

The capacitor voltage (*C*_4_ = 39.566*10^-6^F) is discharged to the load (*R*_0_ = 112.5). Figs 20–23 depicts the voltage and current characteristics for (*C*_*1*_, *C*_*2*_, *C*_*3*_, *C*_*4*_). In *C*_*1*_, the voltage stands at 195.4V, accompanied by a current of 25.02A. Additionally, *C*_*2*_ exhibits a voltage of 366.6V, with a corresponding current of 28.5A, while *C*_*3*_ shows a voltage of 377.9V and a current of 22.5A. Finally, *C*_*4*_ displays a voltage of 366.6V, accompanied by a current of 21.5A. The blue colour represents the Voltage and current of *V*_*C1*_, followed by orange, yellow, and purple by *V*_*C2*,_ *V*_*C3*,_ and *V*_*C4*,_ respectively. When comparing the values, they align with the theoretical and simulation data.

During this operational phase, the charging of the Capacitor (*C*_*1*_) occurs as it is supplied by the inductor (*L*_*1*_) through the Diode (*D*_*3*_) when the diode is in a forward-biased state. The diode voltage remains relatively constant and closely mirrors the capacitor voltage. Consequently, current flows through the diode, facilitating the charging of the Capacitor (*C*_*3*_). Throughout this stage, a positive current is observed through the diode. It’s noteworthy that capacitors (*C*_*3*_) and (*C*_*4*_) are bypassed by diodes (*D2*) in a forward-biased condition, with diode voltage closely tracking the capacitor voltage. Once again, current flows through the diode, leading to the charging of Capacitor (*C*_*3*_) with a positive current.

**Fig 24:**
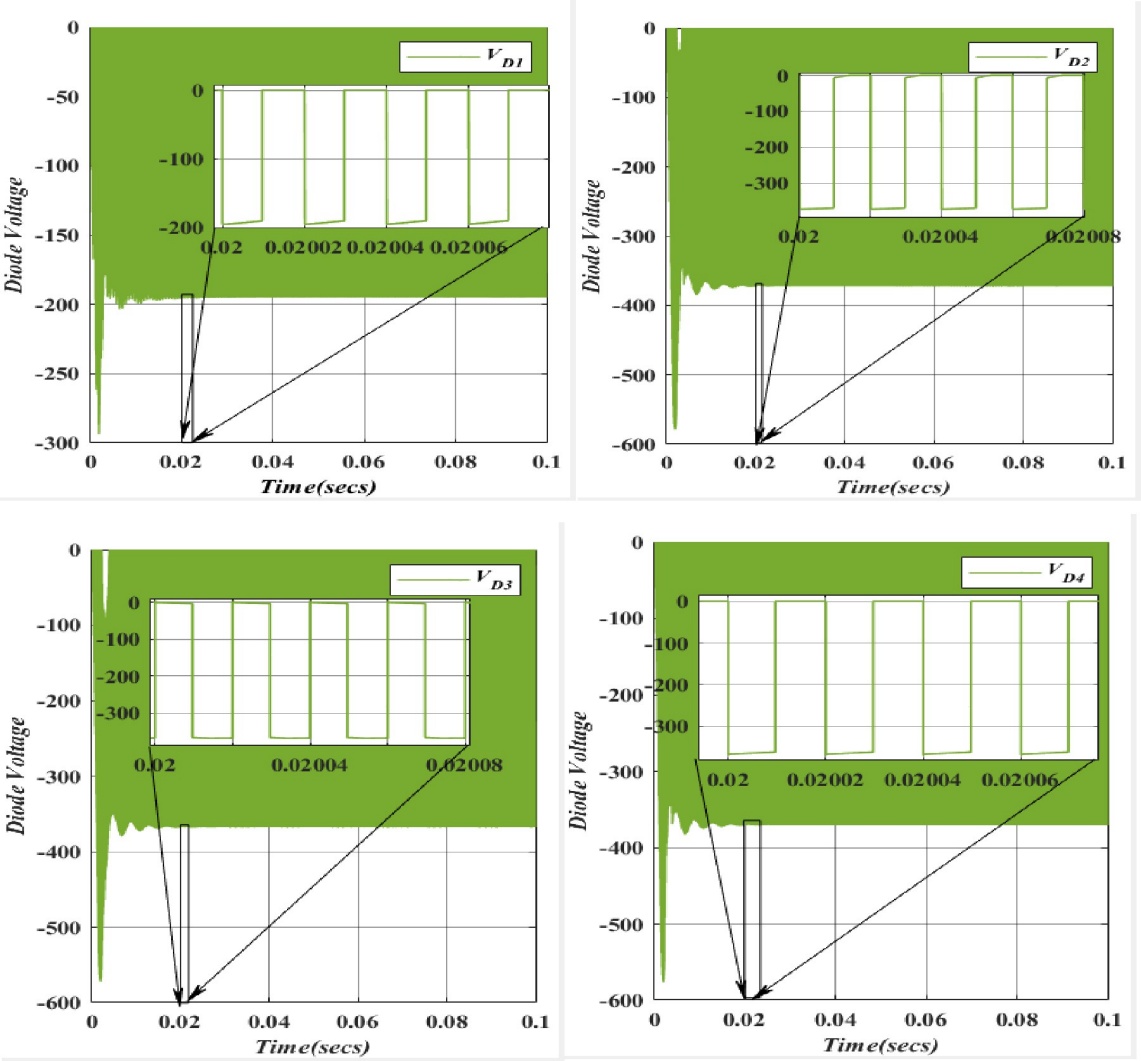
Waveform of voltage for diode (*D*_1_).

In the depicted-ON phase, where the diode is forward-biased, the diode voltage remains relatively constant and closely follows the capacitor voltage. This results in a current flow through the diode, charging Capacitor (*C*_*3*_). Notably, capacitors (*C*_*3*_) and (*C*_*4*_) are bypassed by diodes (*D*_*4*_) in a forward-biased state, with diode voltage closely tracking the capacitor voltage. Once again, current flows through the diode, charging the Capacitor (*C*_*3*_) with a positive current. Fig 24 provides a visual representation of the voltage levels for (*D*_*1*_, *D*_*2*_, *D*_*3*_, *D*_*4*_). Specifically, *D*_*1*_ exhibits a voltage of -93.25V, while *D*_*2*_ displays -180.8V. Additionally, *D*_*3*_ is characterized by a voltage of -189.6V, and *D*_*4*_ shows a voltage of -176.6V. The colour scheme corresponds to the voltages of *V*_*D1*_ (blue), *V*_*D2*_ (orange), *V*_*D3*_ (yellow), and *V*_*D4*_ (purple). These values align with theoretical and simulation results upon evaluation.

Table 3 illustrates a comparative analysis of various converters, underscoring the merits of the proposed converter. The evaluation considered nine different converters, assessing criteria such as voltage gain, energy storage elements, and output values. Among the converters scrutinized is the Step-Up Interleaved DC-DC Converter, High Step-Up DC–DC Converter, A Non-Isolated Buck-Boost DC-DC Converter, Switched Capacitor-Inductor Network based Ultra Gain DC-DC Converter, A Novel Non-Isolated Buck-Boost Converter, Quadratic Boost Converter with Voltage Multiplier Cell, A Non-Isolated High-Gain DC-DC Converter, and High Quadratic Step-up Converter. Detailed results of this comparative analysis are presented in Table 3.

**Table 3:** Comparison among different DC-DC converters.

[25]	**24**	**1.12**	**1.12**	**-**	**47**	**100**	**-**	**-**	**-**	**-**	**109.1**	$\frac{2}{\left(1-D\right)}$
[26] TYPE 1	24	10	10	10	-	100	100	40	20	-	200	$\frac{1+D}{({1-D)}^{2}}$
[26] TYPE 2	24	10	10	10	-	100	100	40	20	-	180	$\frac{1+D}{({1-D)}^{2}}$
[32]	22	0.16	0.6	-	-	24	24	17.8	-	-	110	$\frac{1+D}{({1-D)}^{2}}$
[34]	45	5	15	-	-	100	330	330	-	-	650	$\frac{\left(2-D\right)}{({1-D)}^{2}}$
[35]	30	1	1	1	1	50	50	50	50	470	220	$\frac{2D(2-D)}{({1-D)}^{2}}$
[36]	24	0.15	0.6	0.6	-	220	220	240	-	-	129	$\frac{2}{({1-D)}^{2}}$
[38]	25	0.35	0.65	-	-	220	220	220	220	-	200	$\frac{2}{({1-D)}^{2}}$
**PROPOSED**	100	1.93	3.74	-	-	49.9	26.6	25.8	39.566	-	736	$\frac{2}{({1-D)}^{2}}$

Based on these findings, it can be inferred that the proposed converter is used for wind applications. Unlike other DC-DC converters, the proposed converter offers a higher voltage gain than the alternatives, which utilizes twelve components, including single semiconductor switches, and achieves a maximum gain of 0.48375. Even when compared to the converter referenced in [25], which employs only nine components, the HQSU proposed converter outperforms them in terms of voltage gain. When comparing [26] TYPE 1 and [26] TYPE 2, despite being two distinct converter types, they both yield a similar voltage output with lower gain, each using 12 components. In contrast, the HQSU converter, also composed of 12 components, provides a significantly higher voltage gain. In comparison to the references [34–36, 38,], and [32], while these converters exhibit higher voltage levels than the previously mentioned references, they still fall short of matching the voltage performance of the proposed altered.

The duty cycle and frequency are key parameters in pulse-width modulation (PWM) and waveform generation. Table 4 above compares the different converters’ operating frequencies and duty ratios.

**Table 4:** Comparison of frequency and duty ratio cycle of different DC-DC converters.

REFERENCES	FREQUENCY (*fs*) (kHz)	DUTY CYCLE(D)
[36]	100	0.7
[35]	40	0.5
[38]	40	0.5
[25]	10	(0 ≤ *D* < 0.5, 0.5 ≤ *D* < 1, and 0 ≤ *D* < 1)
[32]	10	0.719
[34]	100	0.27
[26] (TYPE 1)	20	0.6
[26] (TYPE 2)	20	0.6
**PROPOSED**	50	0.48375

Table 5 shows the voltage and current stresses of different DC-DC converters. It is known that voltage and current are interdependent in electrical circuits. Understanding this relationship is crucial for designing and maintaining electrical systems. When there is a small change in voltage it directly impacts current, and vice versa. Higher voltage can increase current stress, potentially affecting components. Balancing both ensures a smooth operation.

**Table 5:** Comparison of voltage stress and current stress of different DC-DC converters.

REFERENCES	VOLTAGE STRESS	CURRENT STRESS
[26] (TYPE 1)	$V_{smax}=V_{C_{1}}+\frac{\Delta V_{C_{1}}}{2}V_{s}-V_{C_{2}}-\frac{\Delta V_{C_{2}}}{2}$	$I_{S(max)}=\frac{(2D+D^{2}+D^{3})I_{0}}{{\left(1-D\right)}^{2}}$
[26] (TYPE 2)	$V_{smax}=V_{C_{1}}+\frac{\Delta V_{C_{1}}}{2}$	$I_{S(max)}=\frac{(2D+D^{2}+D^{3})I_{0}}{{\left(1-D\right)}^{2}}$
[32]	$V_{s1(max)}=\frac{V_{in}}{\left(1-D\right)}$	$I_{S(max)}=\frac{D(1+D)I_{0}}{{\left(1-D\right)}^{2}}$
	$V_{s2(max)}=V_{C_{0}}+V_{C_{2}}=\frac{1+D}{{\left(1-D\right)}^{2}}v_{in}$	$I_{S(max)}=\frac{DI_{0}}{\left(1-D\right)}$
[34]	$V_{s1(max)}=V_{C_{1}}=\frac{v_{in}}{1-D}$	$I_{S1(max)}=\frac{2I_{0}}{{\left(1-D\right)}^{2}}+\frac{V_{0}(1-D)}{4f_{s}}{[L}_{eq}+\frac{\left(1-D\right)}{(2-D)L_{1}}$
	$V_{s2(max)}=v_{C_{2}}+v_{C_{4}}=\frac{v_{in}}{{\left(1-D\right)}^{2}}$	
		$I_{S2(max)}=\frac{2I_{0}}{1-D}+\frac{V_{0}(1-D)}{4f_{s}}L_{eq}$
**PROPOSED**	$V_{s1(max)}=\frac{{(1-D)V}_{0}}{2}$	$I_{s1(max)}=\frac{{(1+2D-D^{3})I}_{0}}{{D(1-D)}^{2}}$
	$V_{s2(max)}=\frac{DV_{0}}{2}$	$I_{s2(max)}=\frac{{(1+D)I}_{0}}{D(1-D)}$

## 7. Conclusion

A thorough assessment and analysis of the High Quadratic Step-Up DC-DC converter’s performance have been carried out to enhance its output voltage capabilities. An extensive review of various topologies and numerous converters has consistently reaffirmed that the High Quadratic Step-Up DC-DC converter finds applications across various fields. This involves detailed investigations into individual converters and comparative studies to determine the most suitable converter for wind turbines. Additionally, the paper delves into the role, challenges, and prospects of DC-DC converters in the context of wind turbines. It also offers an in-depth examination of each multistage converter’s advantages, disadvantages, and specific applications.
